# Drone-Based Digital Phenotyping to Evaluating Relative Maturity, Stand Count, and Plant Height in Dry Beans (*Phaseolus vulgaris* L.)

**DOI:** 10.34133/plantphenomics.0278

**Published:** 2024-11-28

**Authors:** Leonardo Volpato, Evan M. Wright, Francisco E. Gomez

**Affiliations:** Department of Plant, Soil, and Microbial Sciences, Michigan State University, East Lansing, MI 48824, USA.

## Abstract

Substantial effort has been made in manually tracking plant maturity and to measure early-stage plant density and crop height in experimental fields. In this study, RGB drone imagery and deep learning (DL) approaches are explored to measure relative maturity (RM), stand count (SC), and plant height (PH), potentially offering higher throughput, accuracy, and cost-effectiveness than traditional methods. A time series of drone images was utilized to estimate dry bean RM employing a hybrid convolutional neural network (CNN) and long short-term memory (LSTM) model. For early-stage SC assessment, Faster RCNN object detection algorithm was evaluated. Flight frequencies, image resolution, and data augmentation techniques were investigated to enhance DL model performance. PH was obtained using a quantile method from digital surface model (DSM) and point cloud (PC) data sources. The CNN-LSTM model showed high accuracy in RM prediction across various conditions, outperforming traditional image preprocessing approaches. The inclusion of growing degree days (GDD) data improved the model’s performance under specific environmental stresses. The Faster R-CNN model effectively identified early-stage bean plants, demonstrating superior accuracy over traditional methods and consistency across different flight altitudes. For PH estimation, moderate correlations with ground-truth data were observed across both datasets analyzed. The choice between PC and DSM source data may depend on specific environmental and flight conditions. Overall, the CNN-LSTM and Faster R-CNN models proved more effective than conventional techniques in quantifying RM and SC. The subtraction method proposed for estimating PH without accurate ground elevation data yielded results comparable to the difference-based method. Additionally, the pipeline and open-source software developed hold potential to significantly benefit the phenotyping community.

## Introduction

Dry edible beans (*Phaseolus vulgaris* L.) is one of the most significant legume crops consumed directly by humans worldwide because of its major role in nutrition, sustainability, climate change mitigation, food security, and income generation throughout the developing world [1,2]. Dry beans are also an important U.S. specialty crop, with 80% of the dry bean production produced in North Dakota, Michigan, and Minnesota. Dry bean breeding programs work with up to 10 different market classes to develop high-yielding cultivars with desirable agronomic and quality traits, disease-resistant cultivars with appropriate maturity and uniform dry down, upright architecture for direct harvest, and good canning quality [3].

Developing dry bean varieties with appropriate maturity is essential for proper adaptation to a particular target population of environments. As a short season crop that takes 85 to 100 d to maturity on average, dry beans are susceptible to various agronomic and environmental stress that can negatively impact both dry bean yield and seed/canning quality [1]. Varieties that mature too late may be damaged by frost, clog up harvesting equipment, or prove un-harvestable due to rain, while those that mature too early may not maximize yield. Harvest-aid chemicals offer bean growers a convenient tool to hasten harvest by eliminating green stems, leaf retention, and weeds to increase the efficiency of direct harvest. However, preharvest herbicide treatment has been found to negatively affect canning quality by influencing bean color retention [4]. Wallace et al. [5] suggested that indirectly selecting for 3 major physiological components of yield, namely, biomass, harvest index, and days to maturity, should result in improved yield. Therefore, days to maturity or relative maturity (RM) is a crucial trait for dry bean breeding programs to select varieties with appropriate maturity and uniform dry down, reducing the reliance on chemical desiccants and negative effects on canning quality and environmental concerns consumers have regarding residual levels of herbicides in food products.

Plant architecture is the single most important characteristic after maturity that is targeted by plant breeders [6]. Plant architecture has been a crucial trait for dry bean production in the United States to reduce the damage caused by biotic stress, and can also be used to provide natural avoidance for disease infestations, as well as optimize planting density, irrigation, nutrient management, and light interception [7]. Additionally, accurate information on plant height (PH), along with other architectural traits and spatial distribution of plants within a plot, can help breeders to select and develop new dry bean cultivars with improved plant habits (determinate or indeterminate) and plant growth types (i.e., bush, upright vine, and prostrate vine) [8]. This can lead to increased yield, better adaptation to specific environmental conditions, and improved resistance to lodging and other agronomic stresses [9]. Therefore, selecting dry bean cultivars with appropriate PH characteristics, such as upright architecture of plant growth (type II), can not only increase the natural plant defense but also facilitate efficient harvest operations and reduce seed losses [10].

Cultivar selection also depends on various biotic stress conditions such as disease resistance. In 2020, Michigan dry bean producers considered root rots, caused by a complex of soil-borne pathogens, as the number 2 disease constraint in dry bean production, causing yield loss of up to 84% under environmental conditions favorable for soil-borne pathogens [11,12]. Furthermore, depending on the soil-borne pathogen, screening and symptoms can vary. For example, symptoms of *Rhizoctonia solani* can cause seedling damping-off and root rot [12]. Therefore, timely estimation of dry bean stand count (SC) at early growth stages provides useful information for important agronomic decision-making analyzing the survival rate after root rot loss due to damping-off, as well as plant population estimation. Thus, tolerant genotypes can be selected under disease pressure across multiple stages of the growing season by determining the final number of emerging plants through visual inspection.

However, traditional approaches to measure RM, PH, and SC at field level are limited to visual scores that are subjective and prone to error, laborious, time consuming, and costly. High-throughput phenotyping (HTP) using unmanned aerial system (UAS), or drones, has emerged as an essential tool in plant breeding, providing fast, accurate, and cost-effective measurements of various breeding traits [13]. By automating data collection and analysis, HTP enables breeders to rapidly identify and select superior genotypes, thus accelerating the breeding process and reducing costs associated with manual measurements [14,15]. In soybean, recent work in both the public and private sector has shown that phenomic and deep learning (DL) approaches can provide a solution and a cost-effective manner to estimate RM in breeding programs [16–18]. Recent studies have also demonstrated the potential of utilizing remote-sensing data and advanced modeling techniques for accurate PH estimation in various crops, such as maize [19], wheat [20,21], sorghum [22], cotton [23], barley [24], and rice [25]. In lentil (*Lens culinaris* Medik.), phenomics has shown to be a feasible alternative tool for phenotyping *Aphanomyces* root rot, caused by *Aphanomyces euteiches* Drechs [26,27]. The integration of image-based phenotyping enables the acquisition of high-quality phenotypic data rapidly, accurately, affordably, and objectively for plant precision phenotyping. Therefore, developing cost-effective phenomic, machine learning, and DL tools that can be integrated into dry bean breeding programs can accelerate genetic gain and address food and nutritional security worldwide.

Traditional methods for estimating RM in agronomic crops using drone-based imagery involve the use of multiple linear regression, vegetation indices, and threshold values [18,28]. Recently, DL approaches, such as convolutional neural networks (CNNs) [29] and long short-term memory (LSTM) networks [30], have been introduced for RM prediction, offering improved accuracy and adaptability over traditional methods [16]. CNNs have also been used to estimate RM using image classification [17], and deep image feature extraction has been combined with shallow image feature information to improve RM predictions [31]. By extracting relevant features from images without relying on predefined vegetation indices, these models are more robust to variations in environmental conditions and genotypes. Nevertheless, DL models to estimate RM have been ignoring the temporal relationship between drone flight dates and environmental conditions [e.g., growing degree days (GDD)]. An intermediate-level feature fusion based on deep neural network (DNN) [32] can add in parallel stream structure that contains the image and environment condition (GDD) feature subnetworks, in which 2 modalities are eventually combined into a joint representation by a concatenation layer. Therefore, the CNN-LSTM model could be improved by adding weather information such as GDD under environmental stress.

Deep CNN models also have been widely applied as a reliable analytical method to detection and counting of target structure in several crops [33,34]. In particular, Faster region-based CNN (Faster R-CNN) has shown promise as an effective method for detecting plant structures and/or counting individual plants using drone images [35–37]. However, there is a limited number of studies in the literature demonstrating the reliability of DL models for the identification of legume crops such as dry beans and soybeans. Therefore, based on the performance of previous studies, SC can be estimated accurately through high-resolution images across breeding experiment fields under different environmental conditions. Additionally, object detection using DL approaches have the potential to outperform traditional techniques based on vegetation indices, thresholds, and image segmentation, such as watershed segmentation (WS) [38,39] and image processing using Python libraries, by offering higher accuracy and resilience to variations in plant appearance and density [33,40].

Image features can also be explored by considering the morphologic plant structure to estimate PH by using digital surface models (DSMs) or point cloud (PC). Both DSM and PC data sources have been utilized to PH estimation, each offering distinct advantages in terms of data quality and precision [41,42]. Drone-based PH estimation has proven to be highly accurate and efficient, enabling breeders to rapidly collect and analyze large-scale PH data [43,44]. However, the accuracy of height measurements from DSMs can be influenced by inconsistencies in ground altitude [43]. Some methods generally require data from a flight before crop establishment, such as soil elevation or a digital terrain model (DTM), to calculate PH using a crop surface model (CSM = DSM − DTM) [44]. Alternatively, ground points can be identified based on the distribution of the height values within the plot and subtracted on a per-plot basis to extract the height of the plot [43]. Therefore, there are several methods that have been proven effective to estimate PH in breeding and large field crops, depending on factors such as data quality, analysis methods, target crops, and environmental conditions.

The aim of this study was to utilize UAS-derived images of dry bean plants in the field to efficiently estimate RM, SC, and PH. An HTP pipeline was developed to estimate these traits at the plot level across large numbers of breeding plots. The study also aims to compare conventional methods with DL models for accuracy assessment and develop user-friendly HTP tools for the plant breeding phenotyping community. Specifically, this study is aimed at (a) exploring the optimal flight frequency, image resolution, and data augmentation for evaluating RM, balancing data quality with resource efficiency, (b) leverage data augmentation and pseudo-labeling techniques to enhance DL model performance and accuracy in predicting phenotypic traits, (c) investigate alternative approaches for estimating PH when initial flight data are unavailable or limited, maximizing the use of available information, and (d) create open-source, app-based, user-friendly HTP tools and pipelines that can be readily implemented and adapted by the broader plant breeding community. By optimizing drone data using DL approaches, this study addresses critical aspects of HTP to estimate target breeding traits. As a result, breeders can make more precise decisions, reduce experimental costs, and accelerate the release of new varieties.

## Materials and Methods

### Experimental design and ground-based data of RM, SC, and PH

A set of advanced breeding lines Black and Navy market class dry bean trials from both standard yield trials (AYT) and preliminary yield trials (PYT) conducted by the Michigan State University (MSU) dry bean breeding program were measured for RM, SC, and PH for ground-truth (GT) notes and aerial imagery data. Data for RM and PH were collected over 3 growing seasons (from 2020 to 2022) at 2 locations in Michigan, USA: Saginaw Valley Research and Extension Center (SVREC) (43°24′14.9″N, −83°41′49.95″W) and an on-farm trial site in Huron county (43°50′42.4″N, −83°15′18.2″W and 43°51′52.7″N, −83°18′15.4″W), while the SC experiment was performed only in 2022 at SVREC. Trials consisted of breeding lines and check cultivars ranging from early (81 d) to late (103 d) and short (24 cm) to tall (68 cm) breeding lines. Additionally, the plant population ranged from 66 to 166 considering the annotations boxes and GT data (Figs. S1 to S3). Entries were planted in 4-row plots of 4.5 m in length and 0.5 m between rows in which the 2-center rows represent the breeding line and the outside rows represent the border. Alleys between the plots were perpendicular to the rows and were 2.3 m wide. All experimental trials were laid out in an alpha lattice design except the PYT in 2020, which was carried out in an augmented block design due to limited seed availability. Each entry was replicated 4 times within AYT and 3 times within PYT, except that the PYT in 2020 had only one replication. Trials received industry-standard seed treatments, fertilization, and weed control applications at recommended rates.

GT visual RM dates were taken by trained dry bean students and technicians visiting plots and manually scoring plots as they matured, eventually assigning a date of maturity to each plot. RM GT data were rated as soon as the earliest maturing varieties in each location began to senesce. A dry bean plot was determined as mature when plants had senesced and reached physiological maturity, pods were dried and exhibited their mature color, and seeds reached a moisture content at which harvesting is possible without considerable damage [45]. Plots were evaluated twice per week, and maturity dates were interpolated when it was clear that a plot reached maturity in between site visits. The date of plot maturity was expressed as the number of days after 2023 July 31, corrected by the planting date for all environments. GT visual SC was measured manually by counting germinated plants with fully expanded unifoliate leaves at the VC (vegetative cotyledon) growth stage. Manual SC was carried out in 2022 at SVREC location in replications 1 and 2 of AYTs only. The limited scope of the SC GT dataset was chosen due to the time-consuming nature of collecting this trait manually. PH was measured with a meter stick from randomly selected plants representative of the visual average PH inside each plot, measuring the distance from the soil surface, avoiding any mounds or cracks in the soil, to the relative tip of the plant. The Android application Fieldbook was used to record all GT data in the field [46].

### UAS platform and image data collection

The UAS imageries were collected using a DJI Phantom 4 Pro v2 (DJI Technology Co. Ltd.) equipped with digital red-green-blue (RGB) cameras (5,472 × 3,648) and sensor dimensions of 12.833 (mm) × 8.556 (mm). Flights were conducted after germination during the VE (vegetative emergence) and VC growth stages to perform SC analysis. RM flights were carried out from the beginning of maturation to complete maturation, overlapping with the time range when RM GT data were recorded. Flights were performed within 1 h of solar noon to limit shadow effects, not exceeding 25 min in total flight duration. In 2020, flights were conducted once per week, while in 2021 and 2022, we flew 2 times per week. The exact number of days between flights slightly varied if weather conditions (rain, wind) prevented flying. The flight frequency in 2021 and 2022 allowed the simulation of the RM data analysis using 6 and 9 flight missions in total for each year. Waypoints and flying routes were automatically generated using the flight planning software DJI GSP Pro. An iPad was used to prepare missions prior to going to the field. The missions were uploaded to the main control board on the UAS before each flight. The UAS traveled a predetermined route during each flight via the software’s autonomous flying mode. To ensure accurate georeferencing of images, permanent ground control points (GCPs) were placed throughout field corners and centers at regular intervals covering the entire field trial area. The GCPs were surveyed with a Global Navigation Satellite System (GNSS) receiver using a real-time kinematic (RTK) correction (Trimble R4 GNSS system, Trimble, Sunnyvale, CA, USA). Experimental plot data and the flight mission settings ranging throughout the year and location are displayed in Table 1.

**Table 1.** Environment details and flight settings used in this study to estimate RM, SC, and PH traits. A total of 5 environments were measured between 2020 and 2022 to estimate RM and PH, while SC was estimated using data from 2022 at SVREC only. Each field site varied by the total number of plots, planting date, number of GCPs, and flight mission settings. Locations in Saginaw (SVREC) and Huron counties, Michigan. Overlap and GSD slightly varied across drone flights.

Site	Plots	Plant. date	Flight dates	GCP	Altitude (m)	Speed (m/s)	Overlap ^a^ (%)	GSD ^b^ (cm)
2020 SVREC	584	Jul 5 and 17	Aug 14, Aug 21, Aug 27, Sep 3, Sep 9, Sep 17	9	36	4	85–85	0.98
2021 SVREC	524	Jul 2	Jul 14, Jul 19, Jul 28, Aug 2, Aug 13, Aug 18, Aug 23, Aug 26, Aug 30	10	20	4	75–80	0.6
2021 Huron	84	Jul 11	Jul 19, Aug 23, Aug 31, Sep 3, Sep 9, Sep 16	8	16	4	75–80	0.4
2022 SVREC	636 (132) ^c^	Jul 1	Aug 18, Aug 20, Aug 23, Aug 26, Aug 30, Sep 2, Sep 5, Sep 8, Sep 13, Sep 16	8	25 (6 , 7, 10) ^c^	2	85–80	0.7 (0.15 – 0.2) ^d^
2022 Huron	132	Jul 8	Aug 18, Aug 20, Aug 23, Aug 26, Aug 30, Sep 2, Sep 5, Sep 8, Sep 13	8	25	2	85–80	0.7

^a^ Average drone settings across flights.

^b^ Average drone settings across flights.

^c^ Dataset and parameters used to the SC pipeline.

^d^ GSD obtained for different drone flight altitude.

### Image data processing and CNN methods

**Fig. 1.**
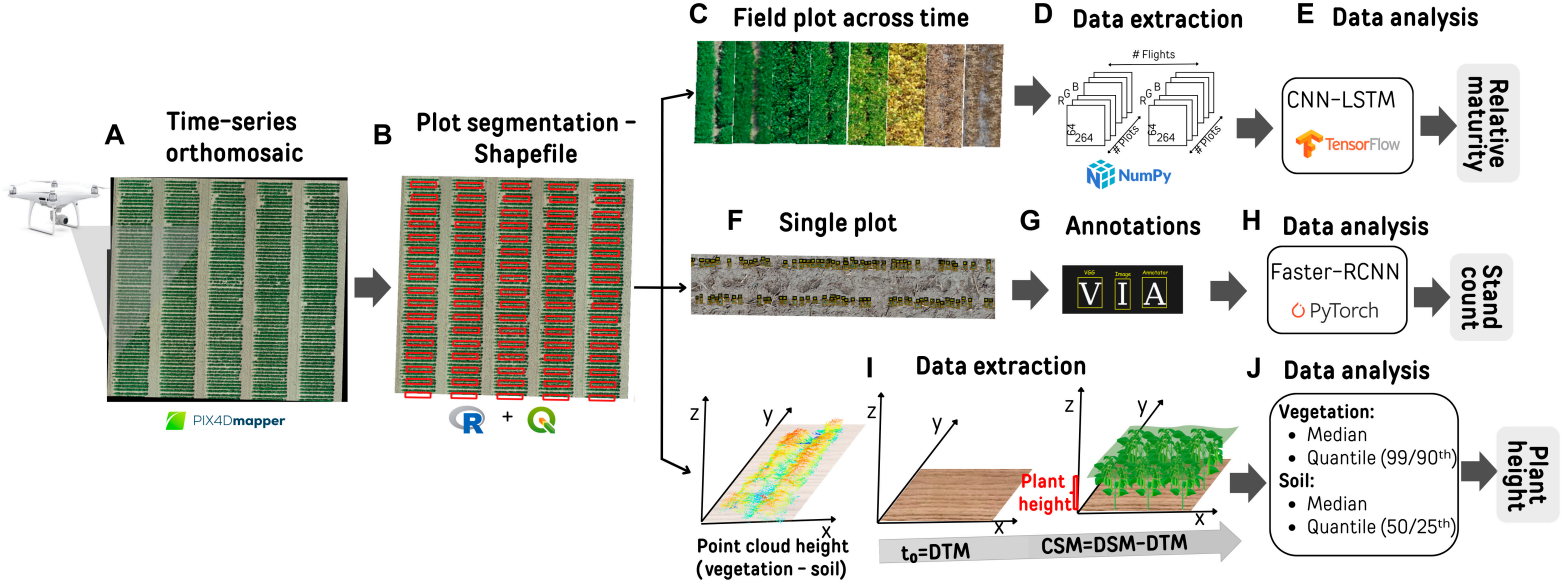
Illustration of the general HTP pipeline for RM, PH, and SC estimations used in this study. (A) Raw JPEG images were captured by an RGB high-resolution drone. Pix4Dmapper software was used to obtain orthomosaics, DSM, and PC data. (B) Shapefile was constructed using the QGIS software integrated with a plugin using the R software. (C to E) Maturity was predicted using a temporal image data collected across the season, and the images were transformed into a high-dimension array using the Python library NumPy to finally use as input in the CNN-LSTM model using the TensorFlow library in python. (F to H) SC was done using single plant annotations and trained using the Faster RCNN model in PyTorch. (I and J) PH was obtained through the DSM (or DTM for bare soil flight) and PC (vegetation and soil flight) performing the subtraction of the elevation from vegetation minus soil using median and quantile to perform pixel value extractions.

The initial steps to obtain the plot-level data were similar for RM, PH, and SC pipelines. The automated pipeline, including photo alignment, matching, and bundle adjustment, were performed using the Pix4D Mapper software (v4.7.5; Pix4D, Prilly) to obtain orthomosaics and DSM (Fig. 1A). The WGS 84 datum was used with a projected coordinate system according to the location UTM (universal transverse mercator) zone. Images were imported into Pix4D and then optimized and matched using pre-established adjusted parameter options for generating georeferenced orthomosaics, DSM, and PC data (RGB Pix4D template at Data S1). The GCPs were input and identified into the Pix4D project using the basic manual editor before initial processing.

The R software (R Core Team, v4.3) integrated with QGIS (QGIS Development Team, v3.32) was used to generate the polygon shapefiles according to plot boundary delimitation using the function “Draw plots from clicks” available at https://github.com/diegojgris/draw-plots-qgis (Fig. 1B). Shapefiles were defined using images collected from the first flight available from each location. GDAL (Geospatial Data Abstraction Library) tool plugin in QGIS was used to spatial polygon vector (or shapefile) adjustments with a buffer zone for each plot to prevent any influence of neighboring plots. Additionally, plot boundary shapefiles were manually aligned as needed to fit on the 2 center rows.

For each flight from the RM pipeline, plot images were cropped from the orthomosaic, and then the data extracted were used to create a time series of plot images. Therefore, each dry bean line is snipped into a single image and then sequenced together by drone flight date. A Python script was developed to efficiently import the orthomosaic, crop the plots using the shapefile, resizing image plot to make the data consistent, and save the image plot using the plot ID information and their corresponding number of days after the planting (Fig. 1C and Data S2). SC plots were clipped similarly to the RM pipeline but using 640 × 3,872 plot sizes instead of 64 × 256 or 128 × 512 as evaluated in RM plots.

The temporal maturity date for each plot across time was extracted using the NumPy library in a stacked array format using 5 dimensions, which contain flight date, plot ID, image height, image width, and RGB bands (Fig. 1D). Additionally, the GT data were adjusted as NumPy format to use as input in the model (Data S3). Then, the time-distributed CNNs automatically extract the image features for each plot image and the LSTM recurrent neural networks are used to predict the RM of bean lines given features extracted using Tensorflow in Python (Fig. 1E).

A single plot was used to annotate all visible bean plants using the VGG image annotator [47] drawing a boundary box to perform the SC pipeline in this study (Fig. 1F). Then, the annotated dataset was used to perform the object detection analysis using the Faster R-CNN in PyTorch with ResNet50 as the backbone, which was pretrained on the Microsoft Common Objects in Context (COCO) dataset (Fig. 1H). This type of model is a region proposal-based object detection method, which uses a region proposal network (RPN) that shares full-image convolutional features with the detection network [48].

Two different approaches were compared in the PH pipeline: heights estimated from DSM and PC data source (Fig. 1I and Data S4). In both methods, the output is produced by subtracting the vegetation elevation from the soil elevation. However, when bare soil flights (DTM) neither are available nor have a good image georeferenced correction (primarily for the *Z* axis, [44,49]), the lowest height values from the DSM and PC data distribution within the plot were assigned to represent the ground (soil elevation), while the data at the top values of height distribution were assigned to vegetation (vegetation height). Therefore, PH was calculated for each individual field plot by using quantile and median strategies extracted from the data height values (Fig. 1J).

A GitHub repository was created to store all the R and Python codes used in this research, as well as datasets, developed apps, and useful information and links to replicate all the analyses to estimate RM, SC, and PH. The GitHub page containing all repositories can be found at https://github.com/msudrybeanbreeding, under the sections DryBean_PlantHeight, DryBean_Maturity, and DryBean_StandCount.

### Maturity DL model

In this study, the proposed DL model by Moeinizade et al. [16] was adapted with modifications to improve the results. In summary, the authors developed a hybrid model to extract image deep features using the time-distributed CNNs and capture the sequential behavior of time series data with an LSTM recurrent neural network. As a result, this model can combine both DL approaches that can process multiple data points and sequences to predict an output. More details about this state-of-the-art DL model can be found in the original model description [16]. Additionally, the same loss function was used in this study to improve the model performance with respect to outliers. This involved combining the mean square error (MSE) loss and mean absolute error (MAE) loss in a piecewise manner, according to Huber loss function [50].

#### Maturity CNN-LSTM model design

The CNN-LSTM model used in this study is based on the model described by Moeinizade et al. [16], which comprises 4 time-distributed convolutional layers. However, in order to streamline the model, reduce the number of parameters, mitigate loss, and avoid overfitting, we made adjustments to various aspects. Specifically, we modified the number of filters, and LSTM layers, while fine-tuning the model’s hyperparameters. A grid search to select the best filter number in each convolutional layer was performed using the WandB [51] central dashboard (Fig. S4). WandB is a machine learning operations (MLOps) tool that provides experiment tracking, collaboration, model versioning, hyperparameter tuning, and model deployment to streamline ML workflows and build reliable ML systems. Additionally, we selected the ideal number of hidden units for the LSTM model through a grid search parameter tuning process (Fig. S5). In the CNN component of the model, down-sampling was performed by max pooling with a stride of 2. To prevent overfitting, we utilized the dropout regularization technique function from the Keras Python library, dropping 20% of the input units.

According to the designed model, the output of the last max pooling layer is flattened and used as input features for LSTM layers, which has 256 units that output a vector. Finally, a dense layer with 1 neuron was applied to estimate the RM of each plot. This architecture has a total of 452.497 trainable parameters, which represent a reduction of 47.71% compared to the original CNN-LSTM model implemented by Moeinizade et al. [16]. The network weights are initialized using Xavier initialization [52], and all activation functions are rectified linear units (ReLU), except for the final layer, which employs a linear activation function. The loss function was optimized using Adaptive Moment Estimation (Adam) with a batch size of 16, a learning rate of 10^−3^, and a decay rate of 10^−3^ [53]. The model underwent training for 200 epochs. The detailed structure of the network used in this study, as well as the model properties, is provided in Fig. S7.

#### Data processing and hyperparameters

Before moving on to prediction, the images undergo several preprocessing steps, as illustrated in Fig. 1 (C and D). In the RM pipeline, all images were resized to a fixed width and length as the original size can vary according to the plot shapefile. According to Moeinizade et al. [16], the image features extracted using the proposed DL model are primarily related to color and shape only, with the image size having minimal impact on predictions. However, the authors did not provide any results regarding image resolution. To explore the influence of image size and image features, we compared 2 datasets with varying image sizes of 64 × 256 and 128 × 512. Unfortunately, due to computational constraints, it was not feasible to test an image size larger than 128 × 512. The image sizes were selected to optimize the retrieval of plot image features, such as shape, while maintaining image resolution consistency with the shapefile plot. This ensured that the extracted features accurately reflected the plot data, enabling more precise analysis and interpretation. In addition to the image size comparison, this study also investigated the effect of flight frequency on prediction performance. To this end, the RM prediction pipeline was trained using the flight datasets from 6 and 9 flights for each of the image sizes under investigation. This approach allowed us to assess the optimal image size and flight frequency for accurate RM prediction using the developed pipeline.

The input dataset is composed of image plots merging 5 environments, of which 80% were randomly selected and the rest of the images were used as test dataset to evaluate performance. Since the number of plots varied across environments (Table 1), the input and test dataset were balanced using the Python library scikit-learn [54] to ensure an equal proportion of plot images from each environment. The input dataset was split into train and validation sets randomly, and 10% of the validation set (10% of input data) was used to monitor the training process.

Additionally, 20% of the total image train dataset underwent testing using data augmentation techniques. These included changing the brightness and contrast and making blur images to test the robustness of the DL model. As discussed by Moeinizade et al. [16], it is very important to test the model prediction accuracy due to the variation of images at the time of collection caused by cloudiness and the relative position between the camera and the sun. The brightness and contrast of images were changed adding a constant to each pixel, and the blur image was done using Gaussian smoothing to remove noise.

A search grid over different values of the hyperparameters σ, batch size, learning rate, kernel size, and decay rate was applied to choose the optimal values that minimize errors and improve the model’s performance. The search grid results were also displayed and saved using the WandB dashboard (Fig. S5). σ is the hyperparameter value from the Huber loss function, and the optimum value was set to 0.1 by using another separate search grid (Fig. S6). The hyperparameter models were trained for 100 epochs to determine the optimal weights and model parameters, utilizing the same strategy for separating the training, testing, and validation datasets, as well as the data augmentation method. This hyperparameter optimization process guaranteed a rigorous assessment of the model’s performance and enabled the final prediction model to attain the best possible outcomes. The results were then saved and visualized using the WandB MLOps tool (Fig. S9) dashboard for further analysis and investigation, and the Python code to perform the analysis can be found in Data S5.

The model performance evaluation was measured using the MAE and MSE metrics applying the optimized hyperparameters selected. The RM DL model was implemented in Python using the Keras library with the Tensorflow backend at Google Colab Pro+. Using the grid search optimal parameters selected, the training model took about an hour on an NVIDIA-SMI Tesla T4 GPU to complete the task. A comprehensive Python script to implement the RM pipeline using DL to perform the predictions for each set of flights and image sizes can be replicated, as demonstrated in Data S6.

#### Plant maturity benchmark

The RM DL model was compared against the maturity pipeline developed by Volpato et al. [18], which utilized a local regression model (LOESS) and segmented linear regression model (SEG). To assess the performance of LOESS and SEG methods, the entire dataset available for each environment was used. In summary, the LOESS model is fitted to the RGB color transformation values over time and SEG model fitted multiple linear models to the data for different ranges of the explanatory variable. LOESS combines nonlinear regression with linear least squares regression by fitting linear models to localized subsets of data determined by the nearest neighbor algorithm [55], while the SEG tests for differences between slopes to estimate the break points between the linear models aiming for the identification of the senescence phase curve [28].

A user-friendly R Shiny application (matuRity app) was developed to implement the LOESS and SEG models (see also Data S8). The digital numbers from bands red (*r*), blue (*b*), and green (*g*), extracted from the visible image (raw RGB images), were used to calculate the VI (vegetation index) value greenness leaf index $\left(GLI=\frac{2G-R-B}{2G+R+B}\right)$. The GLI mean values from each individual breeding plot were extracted from the time series of images (6 and 9 flights date), and the RM was estimated using an optimized threshold value of 0.06. To perform the VI extractions from each breeding plot in the field, an open-source Streamlit app in Python was implemented and can be accessed online at: https://msudrybeanbreeding-vegetation-index--vi-extractions-v0-3-9knpzt.streamlit.app/. Additionally, to accommodate user preferences, an R script is available to perform VI extractions analysis (Data S7).

### SC DL model

**Fig. 2.**
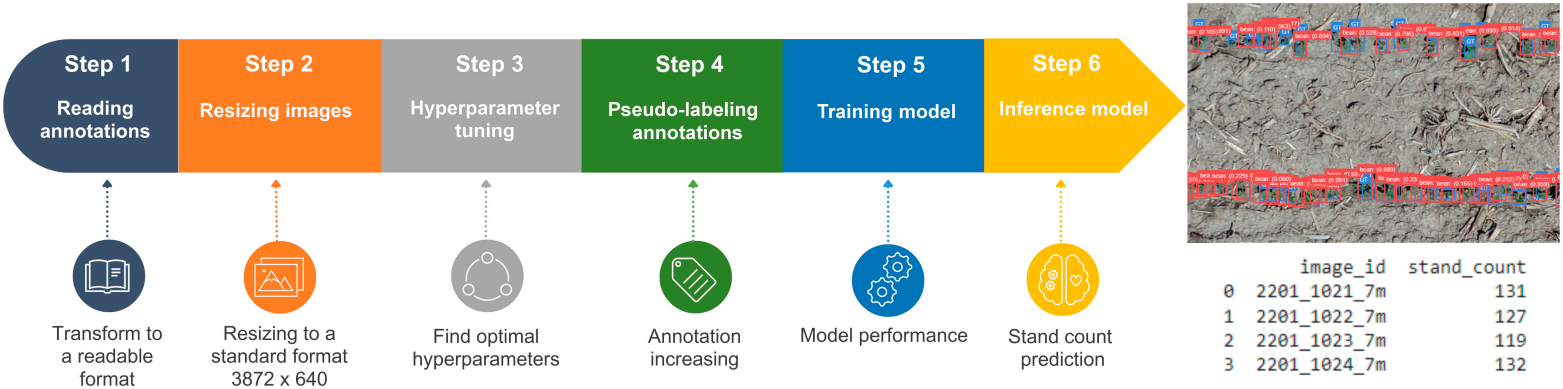
SC pipeline deployed to perform the object detection analysis using Faster RCNN model. First, the annotations were converted to a readable format using the boxes edges (step 1), and in the second step, the images were resized to be ready to use in the training model. Step 3 aimed to find the optimized hyperparameters. Then, to increase the train dataset, pseudo-labeling annotations were performed using the non-annotated experimental plot repetitions 3 and 4 (step 4). Finally, in step 5, the object detection model was deployed using the optimized parameters and annotations (image annotations and pseudo-annotations) to train the model that will be used to perform plant identification and counting in step 6.

The SC pipeline deployed in this study comprised 6 distinct steps, starting from the raw images and annotations, and ending with the final SC predictions. Figure 2 shows the SC pipeline and the detailed description of each step. This pipeline was designed to be user friendly containing all the codes and step-by-step method. In total, 132 field plots were measured as GT and annotated; however, an extra 8 plots were added to the final dataset to increase the annotations. Therefore, in total, 17,259 target single plants were obtained during the annotation step. The VGG annotation tool was used to label the annotated plants from plot images, while the manual data measured at field level were used as GT.

#### SC Faster R-CNN model design

Faster R-CNN is a classical object detection method based on R-CNN and Fast R-CNN, which was proposed by Ren et al. [48]. It combines RPN with the Fast R-CNN module, which makes it possible to acquire feature maps, generate candidate regions, and perform regression and classification operations all fused into one DNN. This method has become one of the most popular object detection frameworks with its advantages of high accuracy and robustness [56]. Compared with the Fast R-CNN, a RPN structure was added in the Faster R-CNN as a representative 2-phase model [48]. The advantage of RPN is the idea of sharing of weights and translation invariance, which not only ensures accuracy but also helps in fast end-to-end recognition [57]. Usually, Faster R-CNN can be divided into 4 parts: a backbone, RPN network, region of interest (ROI) pooling, and fully connected classification and regression model [34]. In this study, the ResNet50-FPN backbone was used taking the amount of computation available into account, as well as the capacity to resolve the degeneration problem of DNNs and generate a high-quality feature map [58]. Additionally, the ResNet-based backbone has enhanced the detection and recognition accuracy on biological targets [59,60].

#### Data processing

The first step in this pipeline, as shown in Fig. 2, consists of reading the VGG annotations and transforming the csv file output to a COCO-style format, which has *x*, *y*, width, and height dimensions (Data S9). Due to the plot boundary (shapefile) variation, some image plots were obtained in slightly different scales. Therefore, in the second step, all plot images were rescaled to a consistent size of 3,872 × 640 pixels (Data S10). The selected image size was chosen to closely resemble the actual plot shapefile size while also meeting the Faster R-CNN image size requirements to maximize the information contained within the plot images.

A grid search hyperparameter was deployed to select the optimum batch size, learning rate, and decay weight values using the WandB MLOps tool (Fig. S8) to save and display the results in a dashboard (Fig. 2, step 3, and Data S11). To this end, the plant detected model was developed using PyTorch 1.8.1 in Python 3.7, and the plot image dataset was randomly divided into 71.5% to training set and 28.5% to validation set. Therefore, the training and validation sets contained 100 images, and the test set contained 40 images. The hyperparameter model was trained for 100 epochs.

To create a valid competition metric, the performance evaluation using the intersection over union (IoU) [61] was used to both hyperparameter tuning and training model steps (Fig. 2). The result of IoU is the ratio of the intersection region of the candidates and the GT to the concatenation region. The ratio is compared with the set threshold, and if the ratio is greater than the set threshold, the candidate box sample is judged to be a positive sample (detected plant); otherwise, it is a negative sample. Additionally, the “Albumentations*”* Python library [62] was also implemented using “OneOf” method to perform a robust image augmentation to increase the quality of trained models. The plot images were transformed using the image augmentation technique pixel-level transform functions: “HueSaturationValue”, “RandomBrightnessContrast”, “RGBShift”, “RandomGamma”, “Blur”, “GaussNoise”, “ToGray”, “RandomFog”, “RandomSizedBBoxSafeCrop”, and “Cutout” [62].

In the grid search hyperparameter training process, the momentum parameter for stochastic gradient descent (SGD) was set to 0.9, and the default IoU threshold between the detection box and the GT was 0.5 and 0.7, respectively. The pretrained weights on the COCO dataset were utilized in the training processing for faster convergence and better generalization [63]. The model training and testing to search the optimum hyperparameters were conducted on Google Colab Pro+ using a Nvidia Tesla V100 graphics processing unit (GPU) with 16 GB video random access memory (VRAM).

Plant recognition using supervised machine learning can be improved by increasing the amount of annotated data to train. Thus, the remaining 124 plots from the AYT in 2022 (repetitions 3 and 4) were used to perform pseudo-labeling annotations to increase the plant recognition accuracy by improving the amount of annotated data to train. This step was done using the optimized parameters selected from the grid search hyperparameter training process. However, to avoid noisy labels due to false-positive detection, the default IoU threshold between the detection box and the GT was set to 0.5 in the inference process. As a result, the jointly box annotated dataset using a total of 264 plots image provided 30,903 recognized plants. These labeling annotated data were used to perform the updated training dataset using the total (264) dry beans plots (Fig. 2, step 5, and Data S12).

The final plant detected model was developed using PyTorch 1.8.1 in Python 3.7, and the plot image dataset was randomly divided into 4 folders aiming 75% or 198 plot images to training set and 25% or 66 plots image to testing set. The optimized hyperparameters in the final training model were adjusted using the SGD with a batch size of 2 [64] to loss function optimization process with a learning rate of and decay rate of 10^−3^ and same momentum parameter and IoU as used to tuning the hyperparameters. The model was trained for 1,000 epochs, and the settings and output were displayed and saved using WandB dashboard (Fig. S13 and Data S13). Additionally, a cross-validation technique was employed to assess the model performance, and the influence of the number of epochs on prediction accuracy was investigated by increasing the epochs to 2,000, which revealed the presence of overfitting (Data S13). Finally, the inference model was performed to classify 2 classes of identified objects, which are plants at early growth stage (bean) and the nonplants that represent the soil (background) using a detection threshold of 0.1 (step 6, Data S14).

#### Plant count benchmark

In this study, the proposed DL object detection model was compared to traditional methods (e.g., handcrafted [35]) using computer vision expert design techniques to extract the plant features via segmentation methods for finding object contours with the OpenCV library [65], as well as the WS approach for detecting pixel intensity values corresponding to regions of an object and determining object boundaries, implemented using the scikit-image library [54], both in Python programming. By integrating the WS approach, we achieved a more detailed and accurate delineation of plant features by refining the segmentation results through the identification of pixel intensity values corresponding to regions of an object. Furthermore, the function “fieldCount” from the R package FieldImageR [66] was adjusted to calculate the number of plants per plot in the software R using a minimum size percentage of plant canopy of 0.01 (Data S17). The OpenCV method, initially developed, follows a series of image processing steps. First, it converts images to grayscale. Next, it applies a Gaussian blur with a kernel size of 5 × 5. Then, it uses canny edge detection with an aperture size of 3 and L2 gradient. After that, it employs the dilation function with a 5 × 5 kernel for 100 iterations. Finally, it applies morphological function closing using a 3 × 3 kernel before identifying contours to determine SCs (Data S15). WS-py method, in contrast, converted images to grayscale, applied Gaussian blur with a 5 × 5 kernel, computed the Euclidean distance map of the blurred image, identified local maxima with a minimum distance of 40 pixels, performed connected component analysis using a 3 × 3 structure, and finally applied the “watershed” algorithm function from scikit-image library with the computed markers and mask to segment bean plants, obtaining SC values (Data S17).

#### Image segmentation

The proposed pipeline to perform SC using the traditional methods (OpenCV and WS) were performed by using a mask obtained from an image semantic segmentation pipeline in PyTorch using a U-Net architecture [67] with a MobilenetV2 as the backbone [68]. In this case, 2 classes were designed, which are soil and vegetations. The loss function was optimized using Adaptive Moment Estimation (Adam) with a batch size of 3, a learning rate of 10^−3^, and a decay rate of 10^−4^. The input dataset was composed of annotated images (140), in which 80% were randomly selected and the rest of the images were used as test dataset to evaluate performance. The input data were split into training and validation sets randomly, and 20% of the validation set (20% of input data) was used to monitor the training process. The image augmentations [62] and the normalization image (using the Normalize function from the PyTorch library) were applied to the images, incorporating the mean and standard deviation values. The model was subsequently trained for 15 epochs.

The semantic segmentation approach was first trained using images segmented via a vegetation index ExG (Excess Green) − ExR (Excess Red). The index derived from the RGB digital number formula $\left(\left(2*g\right)-r-b\right)-\left(\left(1.4*r\right)-g)\right)$, where *r*, *g*, and *b* are the digital numbers from bands red, blue, and green, respectively, extracted from the visible image (raw RGB images). Subsequently, image binarization was done considering soil as value 0 and vegetation as value 1. It is worth noting that SC using traditional methods can be accomplished with binarized images. However, for the purposes of artificial intelligence (AI) using DL models applied to HTP, as well as providing a model that does not depend on a mask (in this study, ExG − ExR) and a threshold to separate soil (background) and vegetation (plants) [69], semantic segmentation can be employed in the future to deliver high accuracy for pixel-level classification. This approach is demonstrated in this study (Data S18) and has been utilized in other studies [70,71]. Evaluation metrics using the mean IoU and pixel accuracy were 0.938 and 0.9967, respectively, providing reliable results for distinguishing between soil and plants (Fig. S11). Consequently, in this study, a DL model was employed to perform pixel-wise segmentation, effectively classifying plot images into soil and vegetation categories.

### Plant height

The estimation of PH (cm) was performed using a quantile method over 2 different datasets: the DSM and PC. The quantiles used to select reference pixels for soil were 0.25 and 0.5, while the vegetation elevation parameters for the plant surface were set at 0.9 or 0.99, depending on the method applied (Table S1). In this study, the same set of flights used to perform the RM analysis was deployed in the PH pipeline. However, it is important to note that the GT measurements were not collected on the same flight days. Instead, we selected the most recent flights performed in proximity to the GT notes to perform this analysis. Therefore, all the flights performed during the measurements in the week were used to get the drone PH results. As a result, the final PH estimations were obtained taking the average of 2 or 3 estimated PH values across flights.

#### DSM and PC generation

The acquired images were processed using Pix4Dmapper to generate a DSM and a high-density 3-dimensional (3D) PC using structure from motion (SfM) algorithm [72] and multi-view stereo (MVS) [73] algorithms to reconstruct the 3D structure of the scene. The PC was later meshed via an algorithm based on Delauney triangulation [74] computed on multiple image scales with noise filtering and a “sharp” surface smoothing filter. The DTM or bare soil elevation was generated for 2021 trials only from images collected by a single flight prior to the vegetation emergence. For all trials in this study, the DSM or vegetation elevation was acquired through flights conducted near the physiological maturity stage. Besides the Pix4D adjusted parameters used in this study, the DSM and DTM rasters, as well as the PCs were computed following the parameter workflow recommended by Pix4D for high-resolution RGB imagery [75]. To calculate the PH using drone images, we utilized the CSM generated from the difference-based method, as described by Volpato et al. [44]. In cases where the DTM was not available, strategies involving DSM or PC were utilized instead. These approaches allowed for the accurate calculation of PH, even in areas where the terrain data were limited or unavailable.

#### PH estimation

In 2021, the CSM (i.e., the height of individual plot surfaces) was obtained by subtracting the DTM from the DSM raster. Data were extracted from the ROIs by overlapping the CSM and the shapefile [43,44]. However, in 2020 and 2022 flights, the PH was obtained by classifying the DSM and PC into ground and nonground (vegetation) points using an assigned quantile of the pixel’s distribution for a given plot. Therefore, the assigned pixels or PC elevation data from the vegetation were subtracted from the soil assigned data for a particular plot. Vegetation data were selected by a single value defined by a quantile, while the soil data were chosen by taking the median value from pixels below the threshold given by a quantile (top lower distribution values) aiming to maximize the PH accuracies and to reduce errors. The parameters employed for estimating PH using the DSM and PC approaches varied across the evaluated environments (refer to Table S1). Parameter adjustments were made prior to comparing the PH with the GT measurements in each environment, based on the observed correlations (results not shown).

To simplify parameter testing and experimental plot visualization and enhance the analysis of PH data in a user-friendly experience, an R Shiny application name “PlantHeightR” was developed and can be accessed at https://msudrybeanbreeding.shinyapps.io/PlantHeightR/ or running in a local machine. The PlantHeightR application was created to support researchers and students to improve and integrate the HTP analysis easily into the phenotyping pipeline.

### Performance assessment metrics

The accuracy of the drone-based phenotyping estimations to RM, SC, and PH was assessed by calculating the coefficient of determination $\left(r^{2}\right)$ and Pearson correlation $\left(r\right)$ between the drone-derived and GT measurements. Additionally, the performance of the deployed models to measure RM was done using the MAE and MSE, while PH and SC were evaluated using MAE and root mean square error (RMSE) metrics, which are defined as follows:

$$\text{MAE}=\frac{1}{N}\sum_{1=1}^{N}|y_{i}^{\;\text{pred}}-y_{i}^{\text{GT}}|$$ (1)

$$\text{MSE}=\frac{1}{N}\sum_{1=1}^{N}{\left|y_{i}^{\text{pred}}-y_{i}^{\text{GT}}\right|}^{2}$$ (2)

$$\text{RMSE}=\sqrt{\frac{1}{N}\sum_{1=1}^{N}{\left|y_{i}^{\text{pred}}-y_{i}^{\text{GT}}\right|}^{2}}$$ (3)

where $y_{i}^{\text{GT}}$ denotes the GT measurements for the ith plot, $y_{i}^{\text{pred}}$ is the predicted or estimated value, and N is the total number of observations.

The evaluation of the Faster RCNN models for the purpose of dry bean plant detection and counting also included precision (P), recall (R), F1-score (F1), and accuracy (Ac). The calculations of P, R, F1, and Ac are shown in Eqs. 4 to 7.

$$P=\frac{\text{TP}}{\text{TP}+\text{FP}}$$ (4)

$$R=\frac{\text{TP}}{\text{TP}+\text{FP}}$$ (5)

$$F1=2\times \frac{P\times R}{P+R}$$ (6)

$$\text{Ac}=\frac{\text{TP}}{\text{TP}+\text{FP}+FN}$$ (7)

In Eqs. 4 to 7, TP indicates true-positive samples, FP refers to false-positive samples, and FN indicates false-negative samples.

Furthermore, a custom algorithm was employed to remove the outlier’s data from PH and SC correlations. This algorithm was also applied to denoise the PC data during the PH analysis to reduce the noise and enhance the overall PC quality. This method utilized the interquartile range (IQR) [76] to identify and filter out the outlier points. Specifically, the difference between the 25th and 75th percentiles (Q1 and Q3) was calculated, and a range of acceptable values were established using a defined multiplier (kout = 1.5) of the IQR. Data points falling outside of this range were considered outliers and subsequently removed from the dataset. This approach facilitated the preservation of approximately >95% to PH and >98% to SC of the original data to all conditions, except to SC at 6-m flight that showed >86% data maintained.

## Results

### RM performance

The DL model used in this study demonstrated high performance in predicting the RM of plots using both 6 and 9 flight datasets across several environments (Table 2). In general, the CNN-LSTM model performed better than the LOESS and SEG models according to the metrics (*r*, *r*^2^, MAE, and MSE) to assess the model accuracy. As expected, the training data results from the CNN-LSTM model showed the highest correlation when compared to the testing data and the performance of the LOESS and SEG methods. This outcome can be attributed to the model’s ability to learn and adjust its parameters specifically based on the training data, ultimately resulting in a more accurate fit for a particular dataset.

**Table 2.** Performance metrics for different models deployed to predict RM in 5 environments (SVREC 2020, 2021, and 2022, and HURON in 2021 and 2022). The DL models (CNN-LSTM) were trained and tested at 2 image sizes (256 × 64 and 512 × 128), and the methods CNN-LSTM, LOESS, and SEG were evaluated using either 6 or 9 flights. The performance metrics measured include *r*, *r*^2^, MAE, and MSE. The metrics are presented separately for the training and test datasets.

		6 flights								9 flights					
Env.	Metric	CNN-LSTM (256 × 64)		CNN-LSTM (512 × 128)		LOESS	SEG	CNN-LSTM (256 × 64)		CNN-LSTM (512 × 128)		LOESS	SEG
		Train	Test	Train	Test			Train	Test	Train	Test		
2020 SVREC	*r*	0.99	0.98	0.99	0.98	0.96	0.96	-	-	-	-	-	-
	*r* ^2^	0.97	0.96	0.98	0.96	0.93	0.92	-	-	-	-	-	-
	MAE	0.54	0.76	0.34	0.76	7.23	6.29	-	-	-	-	-	-
	MSE	0.85	1.21	0.47	1.06	63.84	49.57	-	-	-	-	-	-
2021 SVREC	*r*	0.90	0.24	0.91	0.32	0.16	0.12	0.84	0.28	0.89	0.18	0.20	0.26
	*r* ^2^	0.80	0.06	0.82	0.10	0.03	0.02	0.71	0.08	0.80	0.03	0.04	0.07
	MAE	0.88	2.24	0.82	2.14	5.66	4.11	0.91	2.05	0.70	2.16	5.20	5.50
	MSE	1.55	7.27	1.45	7.03	39.79	24.04	1.90	6.88	1.35	7.18	34.60	37.32
2021 HURON	*r*	0.95	0.82	0.93	0.61	0.87	0.76	-	-	-	-	-	-
	*r* ^2^	0.90	0.68	0.87	0.37	0.76	0.58	-	-	-	-	-	-
	MAE	0.40	1.25	0.43	1.88	7.56	2.94	-	-	-	-	-	-
	MSE	0.46	3.00	0.54	5.38	58.69	16.56	-	-	-	-	-	-
2022 SVREC	*r*	0.94	0.70	0.94	0.79	0.71	0.73	0.87	0.78	0.95	0.79	0.74	0.68
	*r* ^2^	0.88	0.49	0.88	0.62	0.51	0.54	0.76	0.61	0.90	0.62	0.55	0.46
	MAE	0.36	0.98	0.32	0.84	5.59	5.48	0.64	0.91	0.37	0.89	4.56	4.46
	MSE	0.44	1.74	0.41	1.28	35.63	34.03	0.82	1.45	0.32	1.35	23.81	24.74
2022 HURON	*r*	0.90	0.61	0.92	0.80	0.86	0.86	0.83	0.42	0.93	0.49	0.87	0.77
	*r* ^2^	0.82	0.38	0.85	0.63	0.74	0.74	0.69	0.18	0.87	0.24	0.75	0.59
	MAE	0.37	0.96	0.40	0.65	4.31	2.62	0.64	1.08	0.34	1.12	4.12	4.38
	MSE	0.56	1.65	0.51	0.96	19.23	8.69	0.97	1.88	0.41	1.84	17.58	21.23

The error analysis, employing both MAE and MSE metrics, demonstrated a substantial reduction in the CNN-LSTM models compared to the conventional LOESS and SEG methods for all evaluated environments. For the 2020 SVREC dataset, the correlation between GT and DL drone-based image predictions achieved the highest values for the testing dataset (*r* = 0.98 and *r*^2^ = 0.96), outperforming other methods. However, for this specific environment, 2 planting dates were conducted (Table 1), in which the correlation could have been influenced by the variation between planting dates, masking the true evaluation capability of the predictions by extending the range of the response variable. In the 2021 SVREC dataset, the CNN-LSTM models also showed better performance, with the CNN-LSTM model attaining an *r* of 0.32 and *r*^2^ of 0.10 in the test data using 6 flights, which was higher than the LOESS and SEG models, but with larger image data (512 × 128). In the 2021 HURON dataset, the CNN-LSTM model performed well, with the 256 × 64 image size achieving an *r* of 0.82 and *r*^2^ of 0.68 in the test data. Although the LOESS model had a high *r* (0.87) and *r*^2^ (0.76), the CNN-LSTM model showed lower MAE and MSE values, indicating improved prediction accuracy. For the 2022 SVREC dataset, the 512 × 128 CNN-LSTM model demonstrated better performance, achieving an *r* of 0.79 and *r*^2^ of 0.62 in the testing data, surpassing the LOESS and SEG models using 6 and 9 flights. However, the results using 6 flights for both image size showed better correlations and lower errors. The 2022 HURON dataset using the CNN-LSTM model for 6 flights showed better results when compared to 9 flights. Nevertheless, the LOESS and SEG models demonstrated enhanced correlations for 6 and 9 flight sets. However, according to the results in the other environments, the traditional methods presented lower performance in terms of errors compared to the DL models (MAE and MSE <2 for CNN-LSTM models, >17 for LOESS, and >8 for SEG).

Overall, the results demonstrate that the adapted CNN-LSTM models employed in this study were successful in predicting the RM of plots across diverse environments and flight datasets, particularly when comparing errors using MAE and MSE. The performance of the CNN-LSTM models outperformed the LOESS and SEG models, highlighting the potential of DL approaches in practical applications for dry bean maturity prediction.

#### Effect of GDD in the RM prediction

In this study, we selected the 6 flight datasets with an image size of 256 × 64 to investigate the performance of CNN-LSTM model enhanced with GDD data. The decision to use this dataset was based on the balance between the computational cost and model performance, as well as the practicality of data collection in real-world agricultural applications. Moreover, to ensure a fair comparison, the same plots used to create the testing and training datasets were selected for both DL models.

**Fig. 3.**
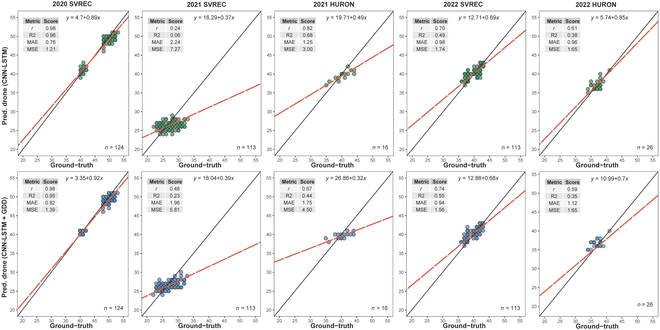
Performance comparison between the CNN-LSTM model and the CNN-LSTM + GDD model across 5 different environments using 6 flights and 256 × 64 image size dataset under 4 evaluation metrics: Pearson’s correlation coefficient (*r*), coefficient of determination (*r*^2^), mean absolute error (MAE), and mean square error (MSE). *n* is the total number of observations in each environment.

The results show that the CNN-LSTM + GDD model outperformed the CNN-LSTM model in certain environments, particularly in 2021 SVREC, where the Pearson’s *r* increased from 0.24 to 0.48 and the *r*^2^ value increased from 0.06 to 0.23 (Fig. 3). Moreover, the MAE and MSE values decreased, indicating better prediction accuracy. This suggests that incorporating GDD information improved the model’s performance, particularly under unfavorable environmental conditions or weather stress.

However, in other environments, such as 2020 SVREC, 2021 HURON, and 2022 HURON, the CNN-LSTM model performed slightly better or similarly to the CNN-LSTM + GDD model. In 2020 SVREC, both models showed high *r* values of 0.98, and similar *r*^2^ values of 0.96 and 0.95. In 2021 HURON, the CNN-LSTM model outperformed the CNN-LSTM + GDD model with a higher *r* value (0.82 versus 0.67) and *r*^2^ value (0.68 versus 0.44). In 2022 HURON, both models showed comparable performances with similar *r* and *r*^2^ values. In 2022 SVREC, the CNN-LSTM + GDD model demonstrated a slightly better performance, with a higher *r* value (0.74 versus 0.70) and *r*^2^ value (0.55 versus 0.49). However, the MAE and MSE values were quite similar between the 2 models, suggesting that the added GDD information did not significantly improve the prediction accuracy in this environment (Fig. 3).

### SC performance

The results demonstrate that the deployed Faster R-CNN model is capable of accurately identifying bean plants at an early growth stage, with a strong correlation between the predicted SC to GT and annotated box measurements. The model performed consistently across different flight altitudes, with relatively low errors when compared to the traditional segmentation methods such as OpenCV and the watershed algorithm. The correlation between annotation boxes and GT measurements (*r* = 0.8) is similar to the correlation between predicted drone values at 7 m and GT measurements (*r* = 0.79). Furthermore, the predicted drone values at various heights (10 m and 6 m) show similar or better performance when compared to annotation boxes (*r* = 0.81 and 0.82 to 10 m and 6 m, respectively). The strong correlation performance observed at a 10-m flight altitude may be attributed to the improved annotation boxes obtained using the 7-m plot image dataset. Therefore, the results indicate that the object detection model employed in this study is resilient under varying flight conditions and can maintain accuracy across different flight altitudes. However, it is important to carefully choose an appropriate growth stage and ensure the use of high-quality annotations.

**Fig. 4.**
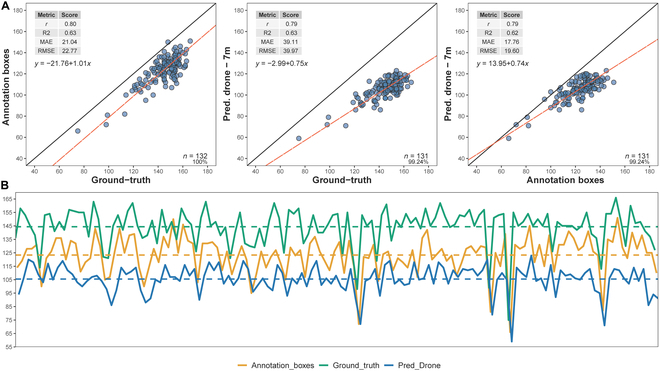
The upper figure (A) presents the performance metrics for the annotation boxes compared to the GT measurements, as well as the predicted drone measurements at 7 m, and (B) shows the trend lines across measured plots to annotation boxes (orange), GT (green), and predicted by drone (blue) to SC.

**Fig. 5.**
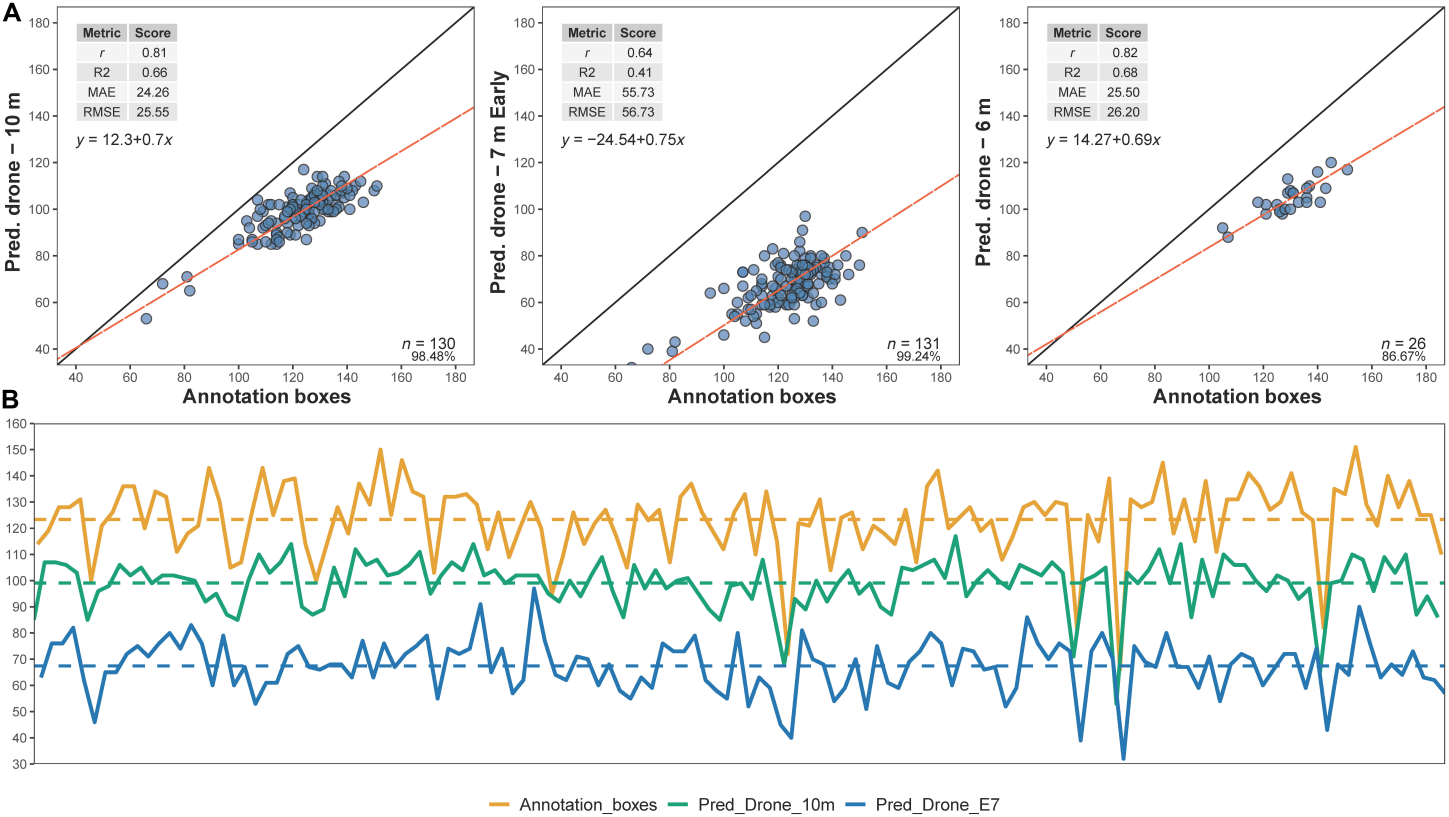
The upper figure (A) shows the performance metrics for the predicted drone measurements at 10 m, 7-m early flight, as well as 6 m compared to the annotation, and (B) shows the trend lines across measured plots to annotation boxes (orange), predicted by drone at 10-m flight (green), and predicted by drone at 7-m early flight (blue) to SC.

Figures 4 and 5 present the results of our study using different flight heights (7 m, 10 m, and 6 m) and various metrics to correlate annotation boxes with SC predictions made by the Faster R-CNN DL model, as well as comparing them to GT measurements. Additionally, a 7-m flight was performed early in the season to assess an early growth stage in the impact on the predictions to detect plants (Fig. 5). Based on these results, the performance of the SC object detection analysis varied with flight altitude, with the drone measurements at 7 and 10 m showing similar results when compared to the annotation boxes; however, the predicted results by drones at 7-m flight showed reduced error as evidenced by the lowest MAE (17.76) and RMSE (19.60) values among the tested altitudes (Fig. 4). The comparison of predicted drone measurements at 7 m with the annotation boxes showed strong performance, though not as optimal as the 10-m flight. In contrast, the early flight at 7 m exhibited a weaker correlation with the GT and higher MAE (55.73) and RMSE (56.73) values. The 6-m flight also demonstrated relatively high correlation and *r*^2^ values (Fig. 5); however, the error analysis (MAE = 25.5, and RMSE = 26.2) presented lower accuracy when compared to that predicted by drone at 7 and 10 m.

The correlation (*r*) between the annotation boxes and the GT was 0.8, with an *r*^2^ value of 0.63, an MAE of 21.04, and an RMSE of 22.77. The predicted drone measurements at 7 m had a correlation of 0.79 with both the GT and the annotation boxes, with an *r*^2^ value of 0.63 and 0.62, respectively. The MAE and RMSE for the predicted drone measurements were 39.11 and 39.97 for GT and 17.76 and 19.6 for annotation boxes, respectively (Fig. 4). The lack of correlations between GT measurements and annotations is also evident in the trend lines shown in Fig. 4. Furthermore, the largest discrepancies in absolute values for the predictions generated by the object detection DL model are observed at the early growth stage, as illustrated by the trend lines in Fig. 5.

#### Comparing SC methods

The DL-based approach, Pred_Faster RCNN, exhibited the strongest correlation with GT SC values (*r* = 0.79) and annotated boxes (*r* = 0.79). The method also demonstrated the highest *r*^2^ for both GT (*r^2^* = 0.63) and annotated boxes (*r^2^* = 0.62). In terms of error metrics, Pred_Faster RCNN yielded the highest MAE (39.11) and RMSE (39.97) for GT among all the methods; however, the Pred_Faster RCNN showed better results compared to OpenCV2 and WS_r when compared to the annotation boxes (Table 3).

**Table 3.** Performance comparison of various SC estimation methods in the context of HTP. The methods evaluated include a DL-based approach (Pred_Faster RCNN), 2 traditional computer vision techniques using OpenCV, and WS algorithms implemented in Python (WS_py) and R (WS_r). Performance is assessed using 4 metrics: Pearson’s *r*, *r*^2^, MAE, and root mean square error (RMSE). The values are reported for both GT SC and annotated bounding boxes.

Method	Metric	Ground truth	Annotation box
		Score	
Pred_CNN-LSTM	*r*	0.79	0.79
	*r^2^*	0.63	0.62
	MAE	39.11	17.76
	RMSE	39.97	19.6
OpenCV1	*r*	0.42	0.49
	*r^2^*	0.18	0.24
	MAE	34.56	15.51
	RMSE	37.38	19.06
OpenCV2	*r*	0.37	0.42
	*r^2^*	0.14	0.18
	MAE	23.43	34.47
	RMSE	28.71	41.75
WS_py	*r*	0.69	0.71
	*r^2^*	0.47	0.51
	MAE	14.87	9.97
	RMSE	17.46	12.2
WS_r	*r*	0.48	0.54
	*r^2^*	0.23	0.29
	MAE	28.43	20.51
	RMSE	35.13	25.44

The WS_py method followed Pred_Faster RCNN in terms of performance, with *r* values of 0.69 and 0.71 for GT and annotated boxes, respectively. The *r*^2^ results for WS_py were 0.47 and 0.51 for GT and annotated boxes, respectively. Also, the method also yielded relatively the lowest error values among the tested methods, with MAE of 14.87 for GT and 9.97 for annotated boxes, and RMSE of 17.46 for GT and 12.2 for annotated boxes.

The traditional computer vision method OpenCV demonstrated lower performance compared to the DL and WS methods. However, the Watershed method in R (WS_r) presented lower accuracy compared to the WS methods in Python (WS_py). In summary, the Pred_Faster RCNN DL-based approach outperformed the other methods in terms of correlation and accuracy, followed by the conventional WS_py method (Table 3).

### PH performance

We evaluated the performance of the quantile method using 2 datasets, CSM/DSM and PC, for estimating PH across 5 different environments. Table 4 summarizes the results, assessing the performance of each data source using *r*, *r*^2^, MAE*,* and RMSE metrics. In this study, the PC data source demonstrated a slightly better performance compared to the CSM/DSM data. The average correlation results across environments were 0.55 for PC and 0.52 for CSM/DSM. The ranges were also similar, with 0.39 to 0.79 for PC and 0.31 to 0.8 for CSM/DSM across different environments.

**Table 4.** Comparison of PH (cm) estimation performance using CSM/DSM and PC data sources across 5 environments. The performance is evaluated using 4 metrics: Pearson’s *r*, *r*^2^, MAE, and RMSE.

	Env.	Metric	CSM/DSM	Point cloud
DSM	2020 SVREC	*r*	0.31	0.39
		*r* ^2^	0.10	0.15
		MAE	8.14	12.93
		RMSE	10.11	15.12
DSM/DTM	2021 SVREC	*r*	0.61	0.63
		*r* ^2^	0.38	0.39
		MAE	20.01	19.15
		RMSE	20.61	19.98
	2021 HURON	*r*	0.80	0.79
		*r* ^2^	0.64	0.62
		MAE	9.14	7.64
		RMSE	9.68	8.29
DSM	20222 SVREC	*r*	0.44	0.48
		*r* ^2^	0.19	0.23
		MAE	19.40	14.32
		RMSE	20.37	15.57
DSM	2022 HURON	*r*	0.47	0.48
		*r* ^2^	0.23	0.23
		MAE	6.38	3.06
		RMSE	7.44	3.83

The average *r*^2^ values for PC and CSM/DSM were 0.36 and 0.33, respectively, indicating a moderate relationship between predicted and observed PH values in both cases. The average MAE values across the 5 environments were 11.42 for PC and 12.61 for CSM/DSM, while the average RMSE values were 12.56 and 13.64, respectively (Table 4). Analyzing by individual environment, the best performance was obtained in 2021 HURON (*r* = 0.8 and *r*^2^ = 0.64) using CSM data and the environment in 2020 SVREC showed the poorest results (*r* = 0.8 and *r*^2^ = 0.64) using the DSM data source.

When comparing the results for estimating PH in 2021 locations using the CSM (derived from DSM – DTM) against the PH solely obtained from the DSM in 2020 and 2022 locations, the correlations between GT measurements and drone-based PH methods demonstrated better outcomes using the DTM for both CSM and PC data sources. The correlations were 0.71 in 2021 locations versus 0.4 in 2020 and 2022 locations for DSM/CSM, and 0.71 in 2021 locations versus 0.45 in 2020 and 2022 locations for PC. However, lower errors for MAE and RMSE were observed in the implemented method that classified the PH distribution into soil and vegetation (DSM only), with an MAE of 14.6 in 2021 locations compared to 11.31 in 2020 and 2022 locations for DSM/CSM, and 13.4 in 2021 locations compared to 10.1 in 2020 and 2022 locations for PC. Similarly, the RMSE was 15.1 in 2021 locations compared to 12.6 in 2020 and 2022 locations for DSM/CSM, and 14.1 in 2021 locations compared to 11.5 in 2020 and 2022 locations for PC (Table 4).

In summary, these results indicate that the PC data source generally showed better accuracy and performance in most cases, although the differences between the 2 data sources were relatively small. The choice between the 2 data sources may depend on the specific environment and flight conditions, as their performance is relatively close in the analyzed scenarios. More detailed information on the PH results can be found in Fig. S12.

## Discussion

The available open-source HTP tools, matuRity, PlantHeightR, and Vegetation index calculator provided in this study, offer researchers a convenient set of tools to extract remote-sensing phenotypes for plant breeding and related areas. The user can either access them online or download the repository at https://github.com/msudrybeanbreeding?tab=repositories. Additionally, the step-by-step pipelines deployed in this study using DL methods are available at the GitHub repositories, as well as the complete dataset used to perform the analysis including orthomosaics, shapefiles, ground notes, clipped plots, and programming codes. Thus, researchers may be able to replicate the present method to predict RM and SC by following the description in this paper along with the scripts developed in Python or R software for each stage from these pipelines. Lastly, the Python-based vegetation index calculator tool can significantly expedite the process to obtain the Vis in large datasets by using as input either orthomosaics or reflectance maps and the field shapefiles. The app also has the option to mask the soil and run the analysis using the selected pixels based on the VIs. The csv output from the app VIs calculator can be promptly used as input data to run the RM analysis at matuRity app.

### Relative maturity

#### Impact of GDD, image size, and flight frequency

**Fig. 6.**
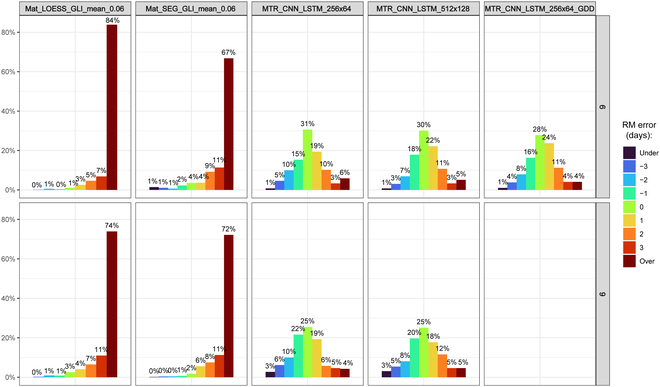
RM window size in days obtained by subtracting GT from the RM estimated across all years and locations of evaluation at 6 and 9 flight frequencies for LOESS, SEG, and CNN-LSTM models. Mean and 0.06 threshold were adopted to perform LOESS and SEG analysis. This analysis can be found in Data S21.

The ability to accurately identify the maturity date or RM of crops is crucial for any plant breeding program as it facilitates data-driven decision-making to deliver better products. Misclassification of dry bean RM can have adverse effects on various aspects, such as harvest operations, agronomic performance, breeding advancement decisions, and overall genetic gain. We have demonstrated that CNN-LSTM provides a robust approach to estimating dry bean maturity date within ±1 d off at 3 locations (2020 SVREC, 2022 SVREC, and 2022 HURON) and ±2 d off at 2 locations (2021 SVREC and 2021 HURON) compared to the actual maturity date in the field without the ambiguity of the LOESS or SEG models due to the setting of arbitrary thresholds and transformation, which lead to information loss (Table 2). In studies performed at soybean trials, CNN-LSTM model results demonstrated comparable performance in their ability to predict RM (±2 d off from GT) [16]. The model performance can also be investigated by comparing RM estimated to the raw data (i.e., GT data). Figure 6 shows in detail the RM estimated error distribution in days obtained when subtracted from the GT data across environments (years × locations) in this study. In both flight frequency scenarios, LOESS and SEG overestimated RM (>66% of RM estimation with more than 3 d of difference to GT), whereas DL models showed high RM precision with 60% of RM estimations within the range of −1 to 1. To predict dry bean RM using a time series of UAS images, color serves as the most important feature, as dry beans mature when their pods turn brownish. Given that color is a simple feature that can be readily detected through the initial layers of CNN, the DL approach employed in this study successfully delivered reliable performance using a straightforward model architecture. Therefore, the proposed study demonstrates that a cost-effective RGB drone implementation can be employed to predict RM without human subjectivity, utilizing image feature extraction through CNNs.

The incorporation of GDD information into the CNN-LSTM model was intended to improve the prediction of RM by accounting for the effect of temperature on drying down the bean trials. The shallow effect has also been used to improve the DL model performance of UAS-based soybean maturity information monitoring [31]. The environmental conditions represent a significant bottleneck in phenotyping [77]. Therefore, there is an increasing need to explore more robust DL models to handle several effects other than image features and also integrates genotyping information [78]. The CNN-LSTM approach serves as a valuable tool for handling feature extraction from both phenotype and genotype input data, providing essential insights into plant characteristics and genetics [79,80]. In this study, the results demonstrated improvements in model performance under specific environmental conditions, particularly when dealing with unfavorable conditions or weather stress (e.g., 2021 SVREC), while in others (e.g., 2020 SVREC), the performance remained similar or decreased. Thus, the model performance demonstrated a potential approach to combine DL image features with GDD-based information to better recognize the environment (Fig. 3). However, the effectiveness of adding GDD information varied across different environments and years. The largest benefit from integrating GDD information was noted in the 2021 SVREC environment, under abnormally hot and dry conditions over a prolonged period of time that hastened maturity (mean values of 10 to 14 d earlier than typical maturity) far beyond typical dry down conditions. This suggests the need for further research and model refinements to optimize the incorporation of GDD and other environmental factors for more consistent performance improvements across various conditions.

Larger image sizes generally provide more detailed information for the model to learn from, which can potentially improve prediction accuracy [81,82]. However, larger images also require more memory and computational power, which may increase training time and the risk of overfitting [77,82]. Comparing the performance of the CNN-LSTM model using image sizes of 256 × 64 and 512 × 128 pixels, we observed varying levels of accuracy across the different environments and flights frequency (Table 2). In general, the 512 × 128 image size yielded better performance than the 256 × 64 image size, indicating that higher resolution images can provide more detailed information for the model to extract relevant features. This improvement in accuracy is particularly noticeable in the 2022 Huron environment when 6 flights were used (Table 2). However, the difference in performance was not always substantial, suggesting that, in some cases, the smaller image size might provide a good trade-off between computational cost and prediction performance.

Higher flight frequency provides more temporal information about the plants’ growth, allowing the models to better capture the dynamics of plant maturity. However, increasing the flight frequency can also increase the cost and complexity of data collection. Our study compared the performance of the CNN-LSTM model using 6 and 9 flights to predict RM. As seen in Table 2, the model’s performance varies across environments and image size. In general, increasing the flight frequency from 6 to 9 flights improved the model’s performance in some environments (e.g., 2022 SVREC at 256 × 64), while the performance remained similar or decreased in other conditions. This suggests that the additional temporal information provided by the increased flight frequency contributed to the improved prediction accuracy in some environments. This finding suggests that an optimal flight frequency could be environment-specific and might depend on factors such as land topography, weather, and soil conditions [16]. However, the gains in performance should be weighed against the additional costs and logistical challenges associated with increasing flight frequency. Therefore, further investigation into the interactions between environmental conditions, drone flight parameters, and capture sensors is essential to achieve an optimal balance between flight frequency and model performance for practical applications in plant breeding HTP pipelines.

### Stand count

#### Plant detection accuracy

Plant recognition using supervised machine learning can benefit significantly from an increased volume of annotated data for training [83,84]. However, obtaining high-quality, consistent annotations is a time-consuming and costly process, often requiring a well-defined multi-stage process involving multiple team members with specialized roles [85]. To expedite the manual annotation process, alternative or supplementary methods, such as transfer learning, weak supervision, or semisupervised learning (SSL), can be employed [86,87]. In this study, we utilized pseudo-labeling annotations, a popular SSL approach, to enhance the amount of labeled data [88]. Pseudo-labeling offers a more efficient means to generate additional labeled data from a fully supervised model, enabling the Faster R-CNN model to benefit from the increased data without incurring the time and resources typically associated with manual annotation [89,90]. By employing pseudo-labeling in our study, we sought to improve the performance of the Faster R-CNN model for SC analysis while maintaining efficiency and minimizing the manual annotation burden.

The reliable performance for the SC pipeline depends on choosing the appropriate growth stage, as well as training dataset quality. In this study, the 7-m plot images were utilized to train the Faster R-CNN model, demonstrating comparable performance in predicting SC not only at 7-m but also at 10-m flight altitude. We found that low image resolution images hinder the visual plant identification and label. However, the training could be done using the high-resolution and low-resolution image dataset to increase the model’s predictive performance. Future directions to increase the accuracy of the model can adopt training dataset increase techniques such as synthetic high-resolution domain using bicubic interpolation or super-resolution techniques [36]. Since Faster RCNN tends to identify objects with similar sizes to the training datasets, it is important to maintain consistency in resolution and plant size between the training and application datasets. Therefore, under limitations to manually perform annotations, high-resolution images must be used in order to improve the plant features identification, as well as data augmentation techniques to deal with images having different resolutions [36].

The results obtained from the 7-m flight dataset using the Faster R-CNN model demonstrate its efficiency and accuracy in identifying bean plants at an early growth stage, with a P of 0.9374, an R of 0.8019, an F1 of 0.8641, and an Ac of 0.76. With a TP rate of 76.1%, the model effectively detects the presence of plants while maintaining a low FP rate of 5.08% (Fig. S13). This low rate of FP indicates that the model seldom misidentifies nonplant objects as plants [91,92]. Additionally, the FN rate of 18.8% demonstrates that the model misses a relatively small proportion of actual plants in the field. These results provide similar performance as those reported by Velumani et al. [36] who obtained an accuracy of 0.88 and precision of 0.95, and also by David et al. [35] with Ac ≈ 0.8. In this study, we employed a GSD (ground sample distance) of 0.15 to 0.2 cm, whereas the cited authors used images with a spatial resolution of approximately 0.3 cm. Karami et al. [93] also obtained good results with Ac of 0.82 with a spatial resolution of around 1 cm. However, it is important to note that these results were obtained from maize plants, which typically have a lower plant population (due to wider spacing between plants in the field) compared to crops like dry beans or soybeans. Therefore, the results obtained in this study indicate the effectiveness of the model in accurately predicting the presence of plants in the field, as well as its ability to identify a high proportion of the actual plants present. Moreover, F1 and Ac confirm its robustness and potential for HTP applications, enabling accurate SC predictions using low-cost RGB drone imagery.

FN plant identification greatly impacted the accuracy of the model, accounting for 18.8% of the total errors in predictions (Fig. S13). Previous studies have emphasized the significant influence of training image resolution on plant identification [36,94,95]. Lower-resolution images display reduced textural information and green fraction per plant, which can hinder the recognition of plant shape or leaf contours due to high variation in leaf appearances, as well as impede plant separation due to leaf obstruction. To address issues related to image resolution when performing SC in crops with high-density populations (e.g., dry beans and soybeans), future research could explore the use of super-resolution techniques [96,97]. These techniques aim to enhance image resolution by adding textural information while maintaining image quality and fidelity [98]. Consequently, employing these methods could mitigate the GSD effect, improve the SC pipeline, and reduce field expenses and resources associated with conducting flights and processing raw images, since fewer images would be required when flying at higher altitudes. A comprehensive error analysis was performed to investigate the overall results, the influence of annotation box size and border annotation boxes, as well as the impact of noisy targets. This analysis can be found in Data S19.

**Fig. 7.**
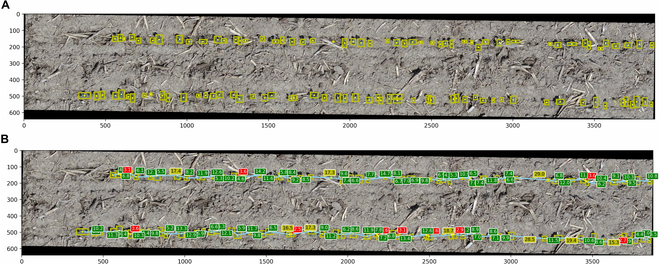
Spatial PD within a dry bean field plot. (A) Bean plant annotated with a centroid point. (B) Distance among the annotated plants in centimeters. In (B), red and yellow boxes depict a poor PD to nearby (<4 cm) and distant plants (>15 cm), respectively, while green boxes show the optimal distribution of plants. The distance between plants is represented in centimeters calculated based on the pixel image size (GSD).

In this study, early flights failed to accurately identify plants in the field due to low image quality and lack of plant features such as leaf shape and green pixels; however, later flights also encountered challenges during the analysis. Leaf obstruction led to a higher rate of plant misidentification (FN error) in drone images. Canopy images may limit plant labeling due to occlusion by leaves, resulting in some plot images with poor plant annotations that lack corresponding lateral texture features, making it difficult for the model to distinguish plants. This situation was also observed even during field-level measurements (GT measurements), as human subjectivity could lead to inaccurate SC data. Consequently, the model was better at identifying plants that were easily labeled and minimally affected by external noise. High plant population density can exacerbate obstruction issues, leading to plant annotations based on the canopy surface. As discussed by Li et al. [34], the inconsistent sizes of rectangular labeling boxes and labeling difficulties could also decrease model performance. The presence of weeds, young leaves, and complex leaf scenes resulting in appearance distortion could lead to detection failures and increased FP ratings [60]. However, under the field conditions examined in this study, our model was not affected by plants other than beans, as the trials were managed under good agronomic practices to control weeds (see Fig. 7 in the next session). This issue could be addressed by including more object classes in the model to identify plants, soil, weeds, and previous crop residue. To achieve this goal, robust data annotations and training images should be used, although it will demand extra time and effort.

The IoU method measures the overlap between the predicted plant bounding box and the GT plant bounding box. In this study, values higher than the 0.5 IoU threshold indicate that the predicted plant bounding box is considered as TP, while values lower than the threshold are FP. Lower values to IoU indicate less accurate plant localization in determining the precise dimensions of dry bean plants; however, an inaccurate estimation of plant dimensions is not critical for those applications assessing emergence rates and uniformity or plant population, where plant density is the targeted phenotypic trait as performed in this study. Consequently, to enhance the performance of the Faster R-CNN model, testing lower IoU threshold values could be beneficial, as demonstrated by Velumani et al. [36], who utilized an IoU threshold of 0.25 to estimate plant density at corn plants. However, the authors also reported that the accuracy of estimating the plant dimension can also be affected by the difficulty of separating the green from the ground in the shadowed parts of the images. When the goal is to evaluate plant size during early developmental stages as well, mask-based RCNN models can be considered as an alternative [99]. Unlike algorithms such as Faster RCNN, which are trained on rectangular regions, mask-based methods can better handle the shadows projected on the ground. However, creating mask annotations can be time consuming, leading to increased efforts in generating a diverse training dataset.

The traditional methods (OpenCV and WS) employ a rational combination of image processing techniques, such as edge detection, morphological operations, distance transforms, and connected component analysis. The Watershed algorithm specifically involves peak detection, while the other methods focus on contour identification [100]. Through the logical integration of these approaches, bean plants can be segmented to enable the determination of SC values. The DL model deployed in this study (Pred_Faster RCNN) achieved an *r* value of 0.79 when compared to GT measurements, while other methods, such as OpenCV, WS_py, and WS_r, achieved *r* values ranging from 0.37 to 0.69 (Table 3).

Traditional methods, such as OpenCV and Watershed (WS), utilize a strategic combination of image processing techniques like edge detection, morphological operations, distance transforms, and connected component analysis. Specifically, the Watershed algorithm focuses on peak detection, while other methods target contour identification [100]. By logical integrating of these techniques, bean plants can be segmented to facilitate SC value determination. In this study, the DL model employed (Pred_Faster RCNN) achieved an *r* of 0.79 when compared to GT measurements. In contrast, other methods like OpenCV, WS_py, and WS_r resulted in correlation coefficients ranging from 0.37 to 0.69 (Table 3). Thus, the object detection model exhibited superior performance compared to handcrafted methods for performing SC in crops of high-density population, such as dry beans. However, traditional methods employing Watershed and image processing with OpenCV show significant potential for achieving satisfactory results when analyzing images from spaced crops, such as maize plants. These conventional analyses can be quite appealing due to their ability to provide accurate results while being less computationally intensive to process.

The discrepancy in the absolute values of the evaluated correlations between GT measurements and annotation boxes highlights the limited association between them, likely due to low-quality measurements in either the field or the annotations. Consequently, lower correlations are anticipated in the predicted analysis (Fig. 4, trend lines). Compared to the image processing methods used in this study (OpenCV and WS), the annotation quality is an important issue when performing object detection analysis [85]. This observation underscores the importance of accurate data collection and annotation to ensure reliable and robust results in object detection studies.

#### Plant distribution assessment

In addition to SC estimation, which can help breeders select resistant varieties based on survival rates, this pipeline can also be utilized to gather information on plant locations within a plot to examine spatial plant distribution (PD). Consequently, it enables the calculation of distances between plants, providing valuable insights for optimizing crop growth and management. This can be taken as a measurement for overall plot and trial quality. For example, assessment of PD can be useful in various applications, including its potential impact on crop yield and overall field management [101]. Uneven distribution in crop population can result in local crowding or insufficient seedlings in the field, leading to a significant decrease in yield [102], as well as create disparities in the light environment within the crop population, impacting crop development [103,104]. Maintaining optimal plant spacing can lead to more efficient resource utilization, such as water and nutrients, and contribute to increased grain yield [105]. Furthermore, optimizing PD can minimize competition between plants, promoting uniform growth and development, resulting in increasing crop yields [106]. In addition to its potential impact on yield, the SC pipeline’s ability to analyze PD can also be used to improve crop models.

Through the application of PD analysis, gaps between plants can be identified, as well as instances where plants are in closer proximity (double or triple plants). Gaining insight into PD patterns can support researchers and breeders in making more informed decisions when selecting traits that contribute to enhanced grain yield and overall crop performance. By incorporating PD data into the prediction model (e.g., use PD as covariate), researchers can produce more precise yield predictions, which in turn can support breeders and farmers in making better-informed decisions related to crop management and cultivar selection. Therefore, the plot images and box annotations used for the SC analysis were also utilized to develop the PD analysis in this study, based on the detected plants and their distances according to the image GSD. As a result, a plot image visualization showing the detected plants and their distances can be obtained (Fig. 7).

In this study, plants spaced less than 4 cm apart were flagged as jointed (red boxes), while gaps between plants greater than 15 cm were flagged (yellow boxes) and annotated, as demonstrated in Fig. 7. The results indicated moderate plot quality, with over 50% of the evaluated plots showing fewer than 10 instances of either closely spaced or widely spaced plants (Fig. S14). Therefore, while reasonable results were obtained using the SC pipeline, the discrepancy between GT and SC predictions could be attributed to the lower quality of the plot fields in terms of plant population and germination/vigor. Implementing this pipeline enables not only SC prediction but also the assessment of PD and germination/vigor in bean breeding trials. However, further investigation is needed to evaluate this model’s performance as an HTP quality control tool. Additional details, result tables, and plot visualizations can be found in Fig. S14, Table S2, and Data S20.

### Plant height

The findings in this study align with previous research indicating that the performance of CSM/DSM and PC for PH estimation may vary depending on the specific environment and flight conditions [43,44]. Overall, the PC data source showed better accuracy in most cases, suggesting that it may be more suitable for PH estimation in HTP applications. However, it is important to consider that the performance differences between the 2 data sources were relatively close, implying that the choice between them may ultimately depend on factors such as data availability, processing requirements, and environmental conditions.

Parameters related to plant morphology and structure, such as PH, can be derived from 3D reconstruction dense PCs, orthomosaics, and DSMs, otherwise known as digital elevation models [43]. These methods utilize SfM algorithms in downstream image analysis, where multiple 2D images are overlapped to generate a 3D reconstruction [107]. The accuracy of PH metrics extracted from SfM PC data is generally superior compared to other approaches [108], a finding that is supported by the results of this study. The estimated PH obtained from the PC dataset showed better performance with higher image resolution (lower values of GSD), as observed in the 2021 experimental trials (Table 1). This is particularly evident in the 2021 HURON trial, which achieved the best correlation between GT and drone-estimated PH across all environments for both DSM and PC methods (*r* = 0.8; Table 4). However, PC data consists of thousands of data points, which can exponentially increase with higher image resolution and overlap, thus demanding more computational resources. This time-consuming analysis becomes evident when users choose to use PC data instead of DSM for PH analysis with the PlantHeightR app. To expedite the process, users can crop the precise field area where PH is being collected (see the “Snipping tool” tab in the app). Nonetheless, as shown in this study, the DSM dataset can be utilized as an alternative to extracting PH instead of using larger PC data, effectively reducing computational demands.

A high-quality DTM or ground altitude is essential for enhancing PH estimations, particularly when the soil elevation contains inconsistencies. There are several methods to determine ground altitude such as DTM interpolation method, difference-based method, manual measurement self-calibration method, and exposed alley subtraction method [43]. The findings of this study demonstrated that the subtraction method [19], which assigns height values within a plot and subtracts them on a per-plot basis to extract the PH, provided reliable accuracy for PH estimation using DSM and PC data source. Nevertheless, to decrease absolute error values, this method depends on the availability of unobstructed areas with exposed soil, which may not always be present in all crops and field layouts. Furthermore, this approach allows for the calculation of ground height for each individual plot, reducing the computational intensity needed for interpolating a DSM across the entire field, as implemented in the PlantHeightR app analysis.

Precision positioning information plays an important role in the accuracy and quality of a DSM [109]. As a result, georeferenced correction is highly recommended for improving PH results, particularly when dealing with UAS systems that have less accurate internal GPS. To compensate for UAS GPS inaccuracies, GCPs with high-quality GPS information can be placed in the field. Acquiring accurate soil or vegetation elevation data can be challenging and resource intensive, particularly in the absence of georeferenced corrections such as RTK/PPK (post-processed kinematic) or GCP at the plot level. This approach can be time consuming, both at the field level and during data processing. The deployed subtraction method in this study showed a good alternative to estimate PH at plot level rather than use the DTM from the difference-based approach. Hence, this method can be employed even in the absence of GCPs, as the analysis relies on the in situ plot PH value distributions. This flexibility allows for more accessible and efficient estimations without compromising the overall accuracy of the results.

The accuracy of PH measurements using drone imagery is also dependent on which pixels or data are chosen to represent either the top of plants or the soil level altitude. Thus, the erroneous pixels can be removed by selecting the optimal SfM metrics for both DSM and PC PH extraction. This process can be done by testing several thresholds and extraction methods for soil and vegetation classification. The optimal metric selection can vary by crop and study design due to several environmental factors such as occluding leaves and wind movement, which can generate SfM artifacts, as well as changes based on growth stage, planting density, and image resolution [21,22]. The PlantHeightR app can easily handle these parameter optimizations, by providing common SfM metrics including the extraction methods mean, median, and percentiles, as well as test different thresholds to vegetation and soil classification (Table S1).

## Conclusion

This study demonstrates the potential of using low-cost aerial images and advanced machine learning techniques, alongside well-established traditional methods, for HTP applications in dry bean trials. The developed pipelines provide plant breeders and researchers with a cost-effective and efficient solution by incorporating RM prediction, SC assessment, and PH estimation. Additionally, open-source software available in this study can support breeding decision-making and streamline HTP pipelines through user-friendly parameter analysis. CNN-LSTM and the Faster R-CNN models for predicting RM and SC, respectively, showed promising results in various flight conditions using DL applications, making them valuable tools for decision-making in breeding programs. The choice of image size, flight frequency, and the inclusion of GDD can have varying effects on the CNN-LSTM model performance depending on the environment. Future research should focus on refining these parameters to optimize RM model performance and further explore the potential of incorporating additional environmental factors, such as soil conditions and weather, to improve the accuracy and robustness of the model. Traditional methods for RM and SC analysis offer reliable alternatives to more complex DL techniques, although their success depends on optimal threshold selection and effective soil segmentation. DL models demonstrated accurate performance in predicting RM with fewer flights and lower image resolution, as well as effective SC prediction in high-density crop populations. The assessment of PD in the field is of great importance for optimizing crop management, improving yield predictions, and ultimately increasing grain yield. These advances in DL technology help reduce phenotypic errors caused by individual subjectivity, leading to more objective and reliable measurements with significant implications in plant breeding. Future research should focus on refining the DL models and exploring additional factors, such as plant health, weather conditions, and management practices, to improve the accuracy and robustness of the predictions. Our findings also suggest that the optimum soil elevation data into PH estimation can lead to improved correlation coefficients, as well as highlight the importance of selecting the appropriate data source for PH estimation at dry beans across different environments. Further research is needed to investigate the potential of integrating multiple data sources, such as CSM/DSM and PC, and leveraging advanced machine learning algorithms to improve the accuracy and robustness of PH estimation across diverse crop species and growth stages.

## Data Availability

Developed software and analysis are available in the GitHub repositories at https://github.com/msudrybeanbreeding and datasets can be download at Zenodo deposit page (https://zenodo.org/) using the links to RM: https://doi.org/10.5281/zenodo.7922565; SC: https://doi.org/10.5281/zenodo.7922584; and PH: https://doi.org/10.5281/zenodo.7922589.
