# Supplementary material for: Drone-Based Digital Phenotyping to Evaluating Relative Maturity, Stand Count, and Plant Height in Dry Beans (Phaseolus vulgaris L.)
Source: Plant Phenomics. 2024 Nov 28;6:0278. doi: 10.34133/plantphenomics.0278 (PMC11602537; doi:10.34133/plantphenomics.0278)
Supplement: Supplementary 1 — Figs. S1 to S14 Tables S1 and S2 Data files S1 to 21 [file plantphenomics.0278.f1.zip › Supplementary Materials_R1.docx]

**Supplementary figures**


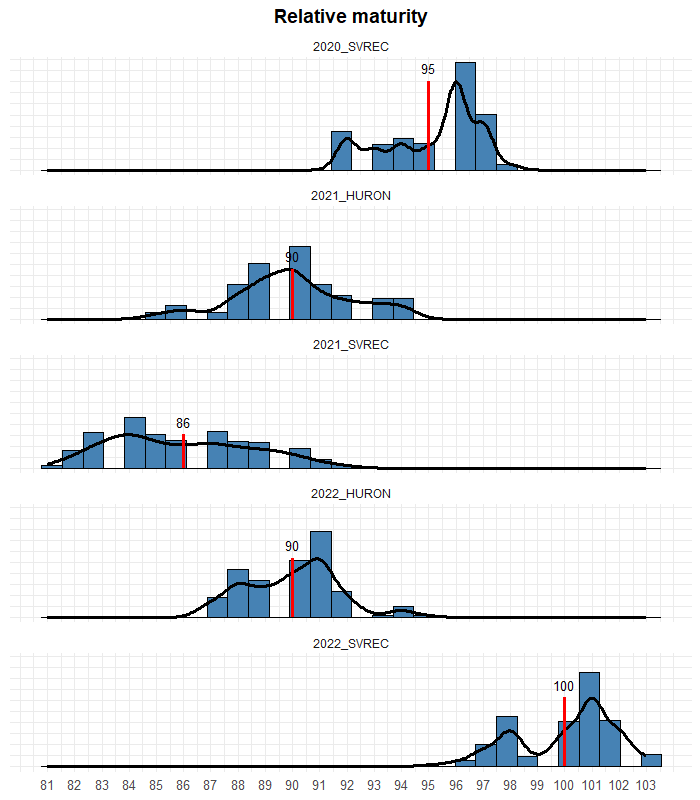


Fig. S1. Histograms of relative maturity (RM, days) distribution for the five environments evaluated. Relative maturity day is defined as days since July 31, corrected by the planting date. The vertical red line represents the average value in each environment.


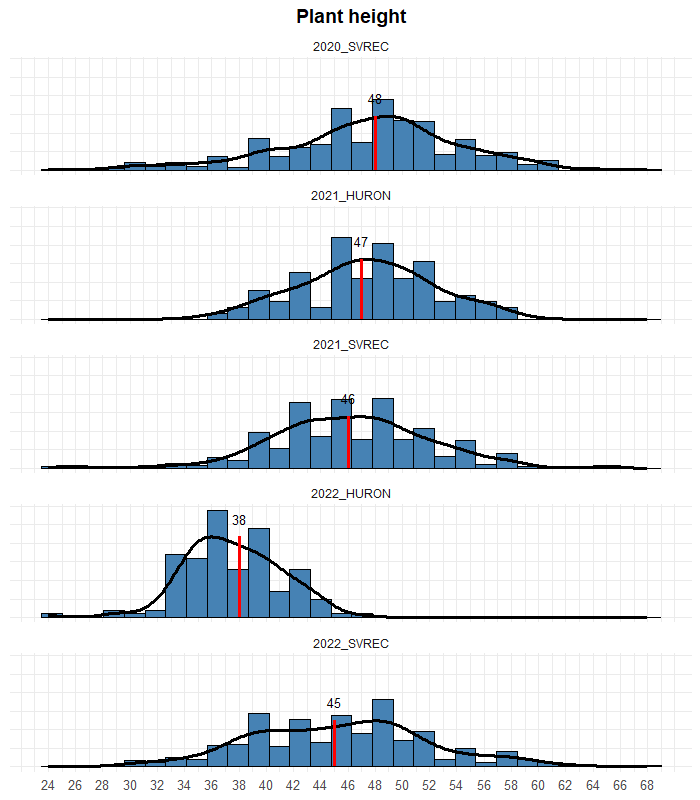


Fig. S2. Histograms of plant height (PH, cm) distribution for the five environments evaluated. The vertical red line represents the average value in each environment.


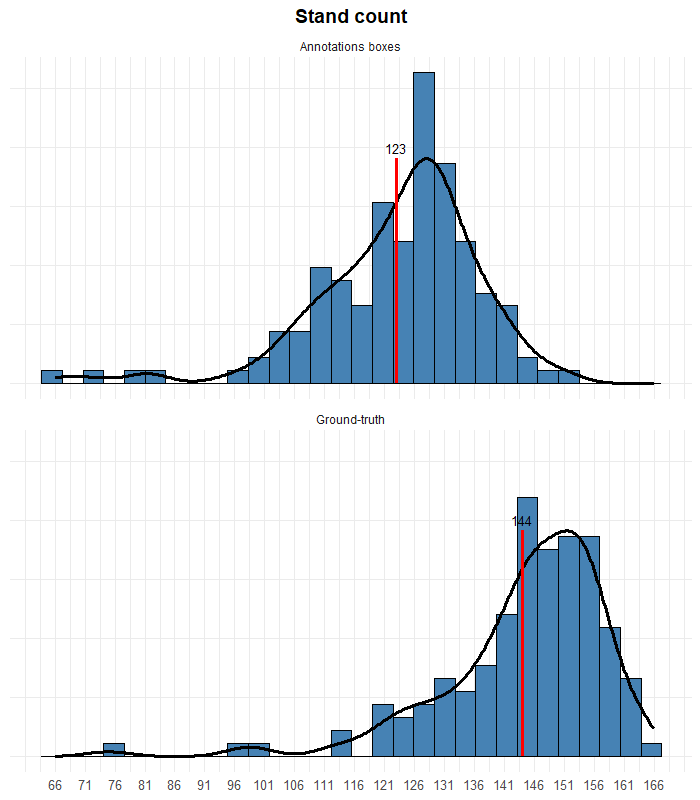


Fig. S3. Histograms of stand count (SC, unit) distribution for the annotated and ground-truth measurements. The vertical red line represents the average value in each data set.


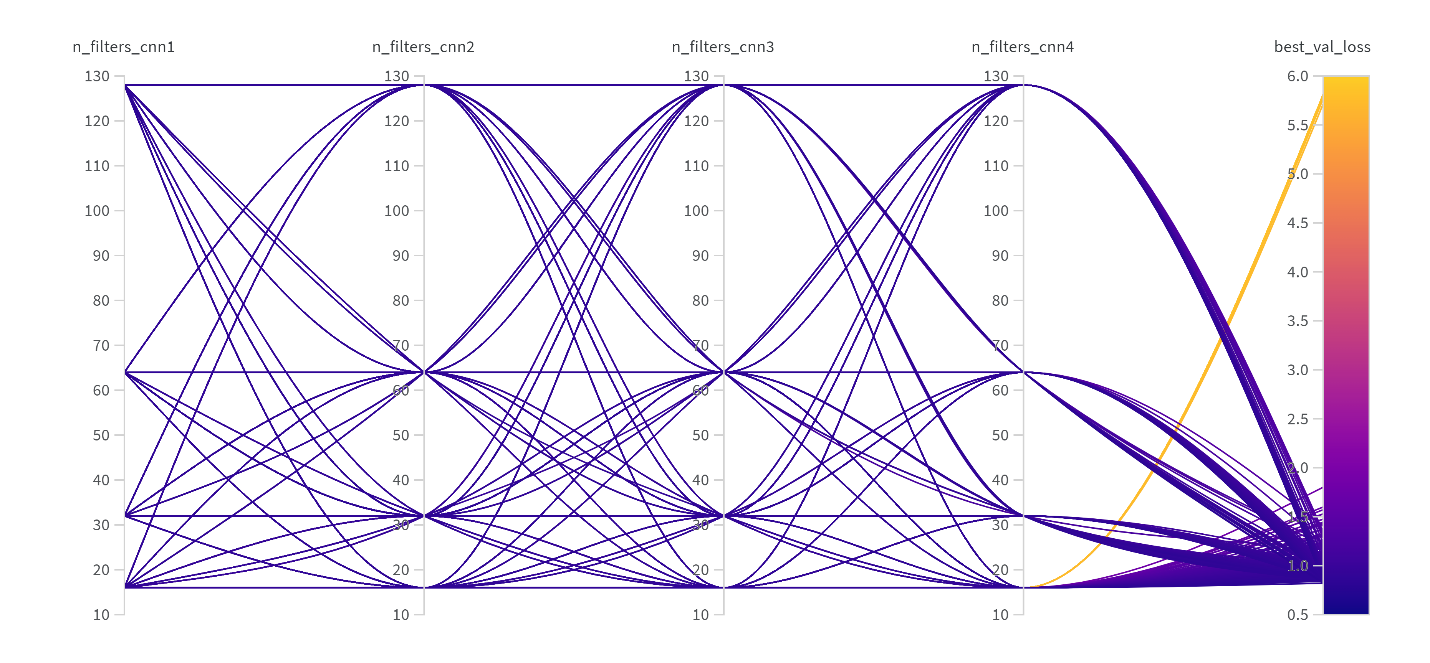


Fig S4. Hyperparameter tuning grid search of filter number. Four time-distributed convolutional layers were adjusted according to the grid search filter number. Results dashboard available at: https://wandb.ai/beanlab/CNN-LSTM-Hyper_MTR_v01?workspace=user-beanlab


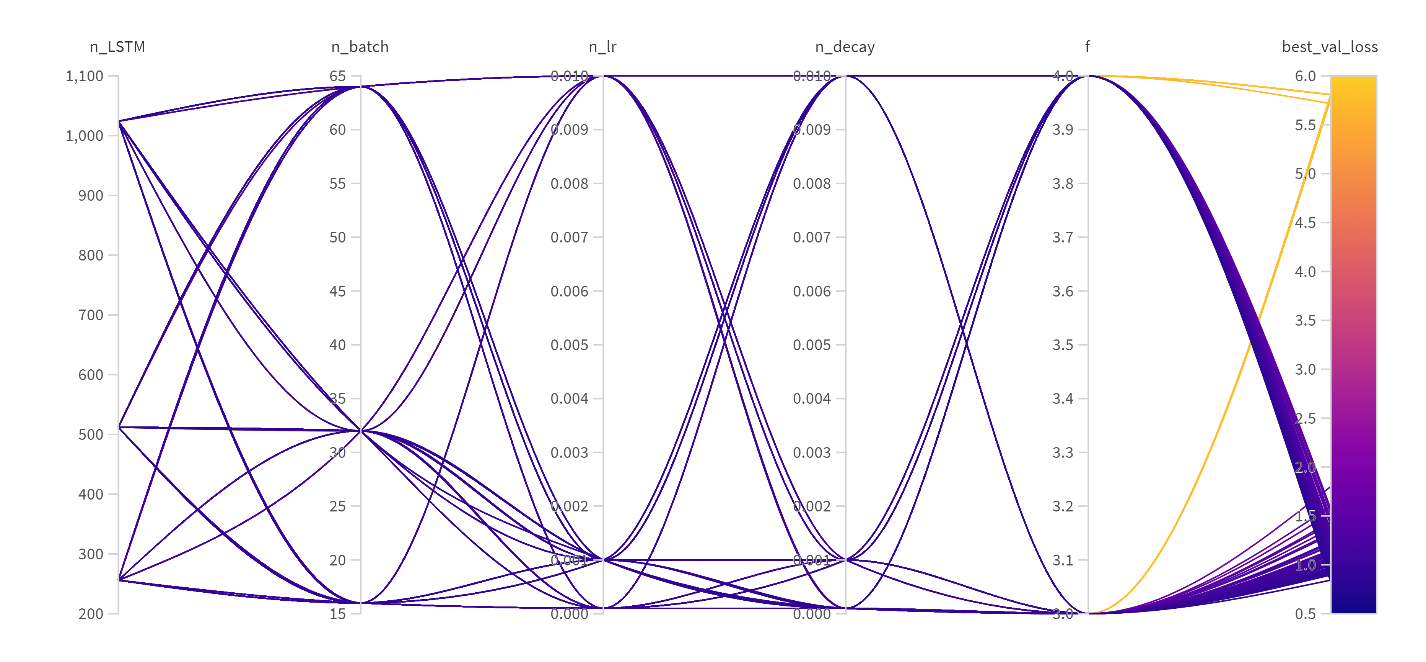


Fig. S5. Hyperparameter tunning grid search of LSTM hidden units, batch size, learning rate, decay rate, and kernel size. Results dashboard available at: https://wandb.ai/beanlab/CNN-LSTM-Hyper_MTR_v01?workspace=user-beanlab


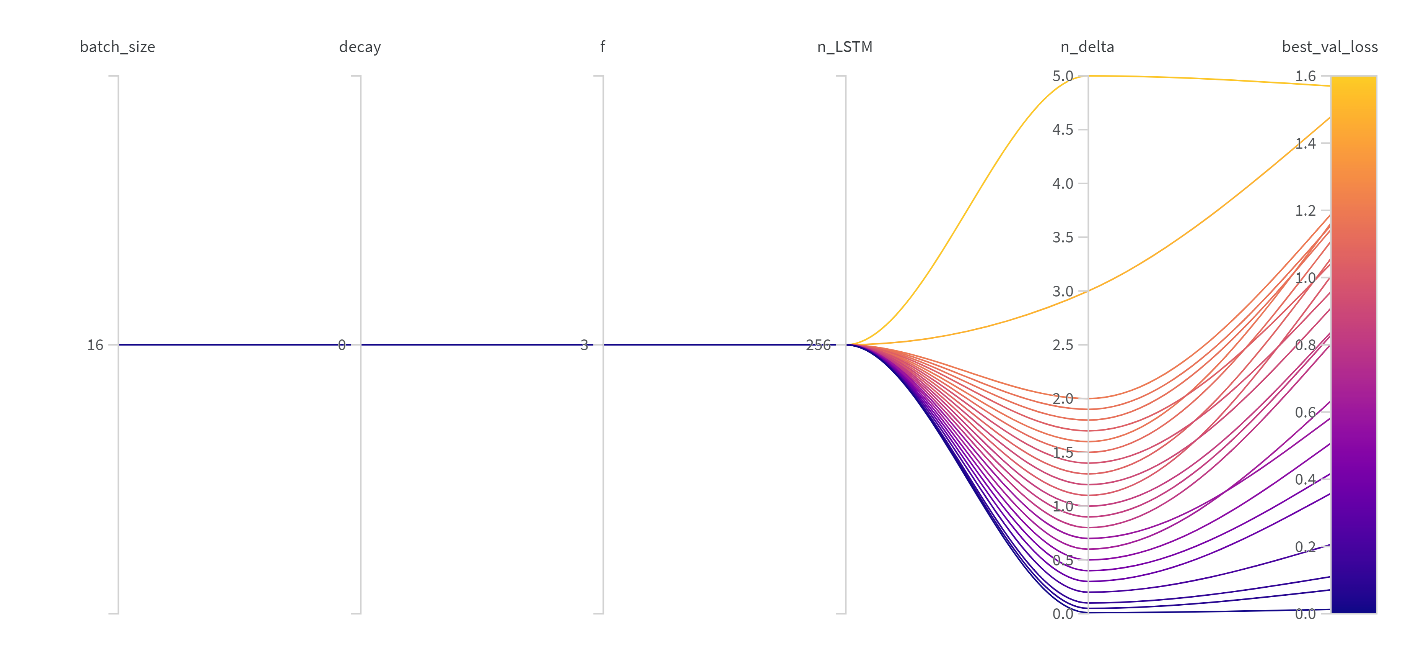


Fig. S6. Hyperparameter tunning grid search values to Huber loss function (σ). Results dashboard available at: <https://api.wandb.ai/links/beanlab/i3lbtx2y>


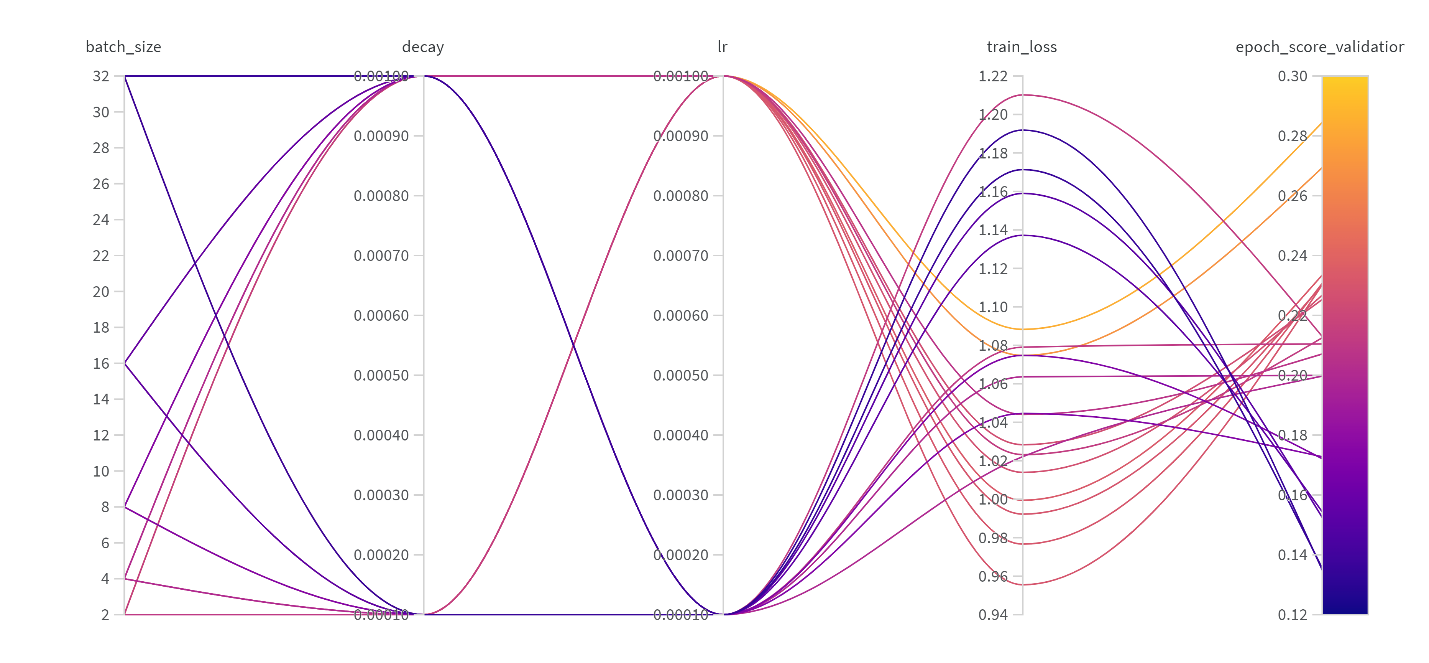
Fig. S8. Hyperparameter tunning grid search value to batch size, decay weights and learning rate. Results dashboard available at: https://wandb.ai/beanlab/BeanStandCount-fasterRCNN_Hyper_v03?workspace=user-beanlab

CNN-LSTM


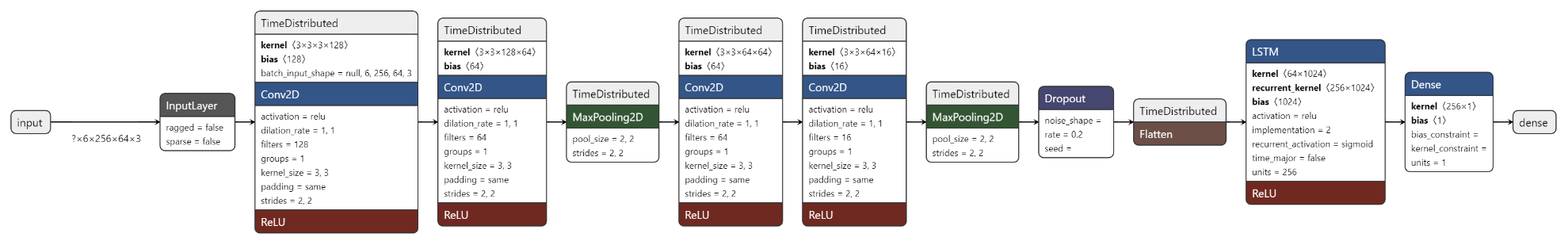


CNN-LSTM + GDD


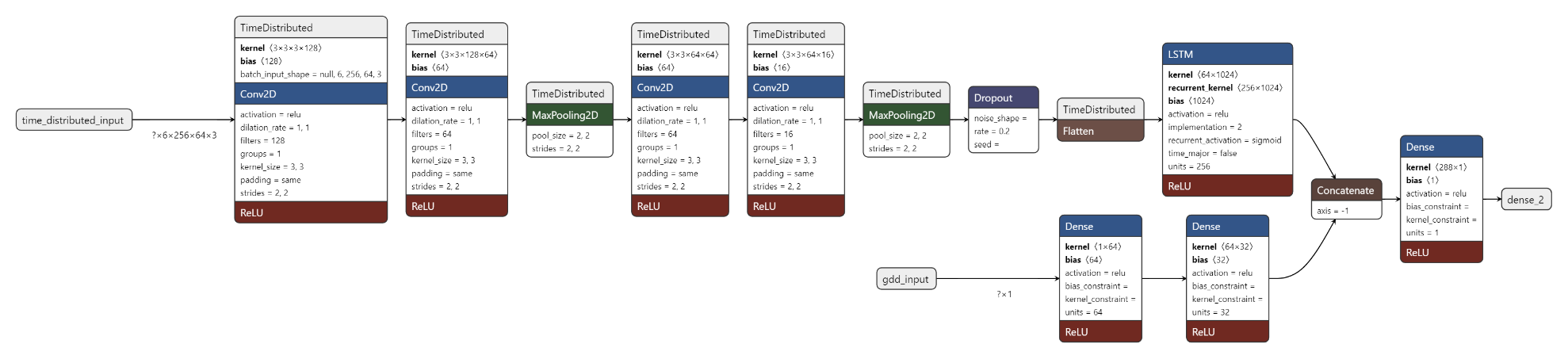


Fig. S7. Detailed CNN-LSTM network models deployed. The GDD was added via intermediate-level feature fusion based on Deep Neural Network. Figure output generated using Netron, Visualizer for neural network, deep learning, and machine learning models (Lutz Roeder, 2017).


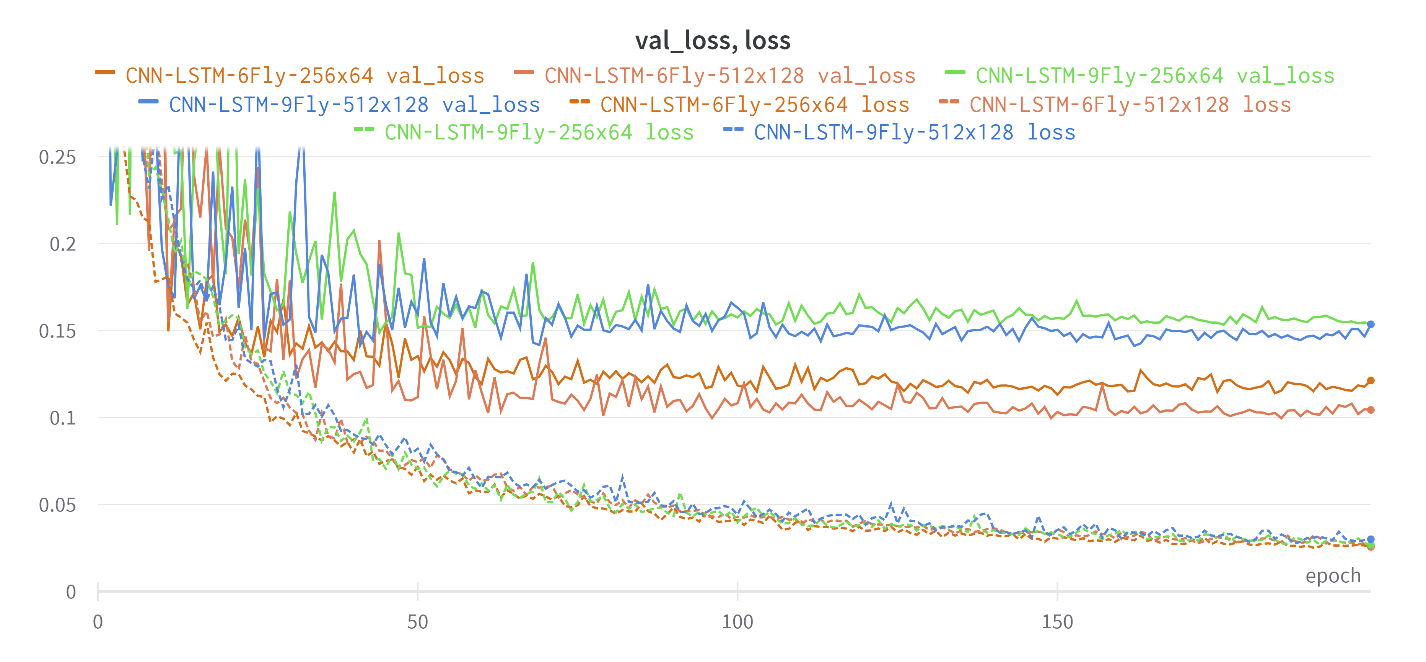


Fig. S9. Validation and training loss CNN-LSTM model performance across 200 epochs for two image sizes (256x64 and 512x128) and two set of flights (6 flights and 9 flights). The WandB dashboard results is available at: https://wandb.ai/beanlab/CNN-LSTM-Train_MTR_v03?workspace=user-beanlab


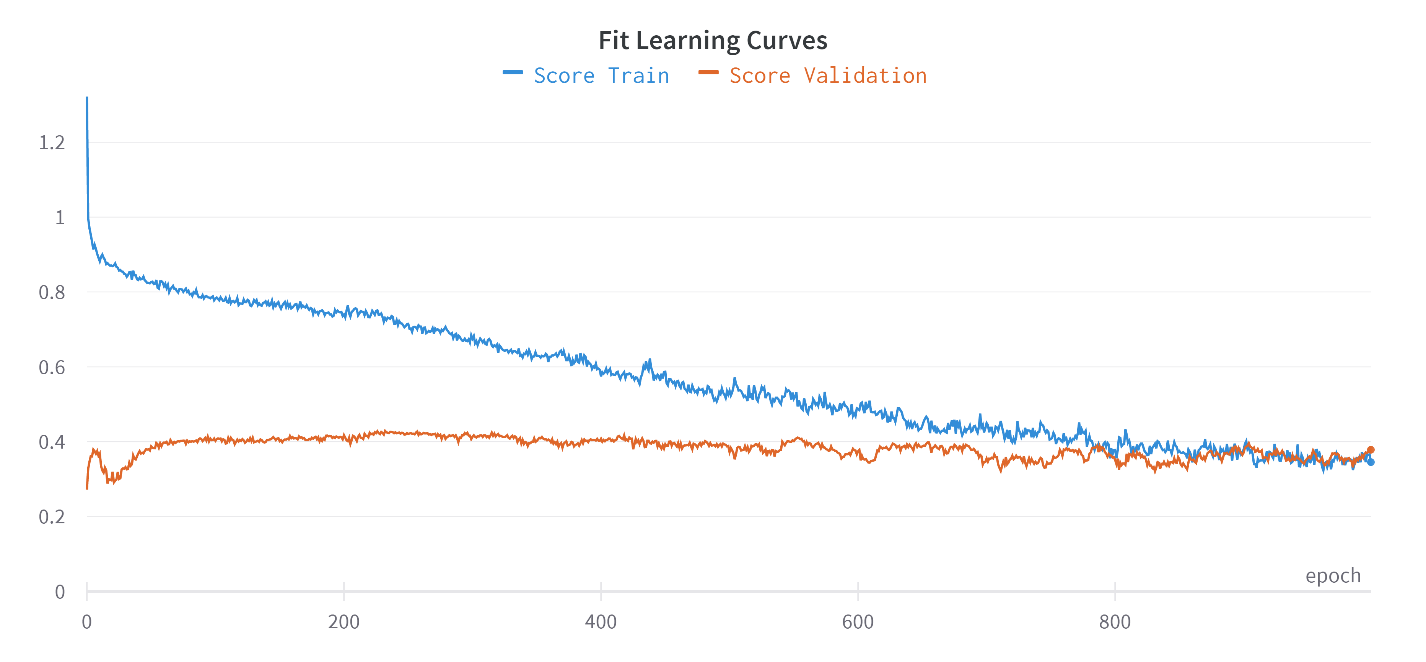


Fig. S10. Validation and training loss fit learning curves for the Faster R-CNN model performance across 1000 epochs. The WandB dashboard results is available at: https://wandb.ai/beanlab/Beans_Stand_Count_final_1000ep?workspace=user-beanlab


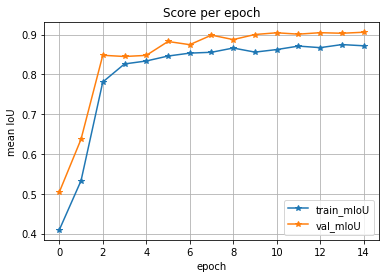

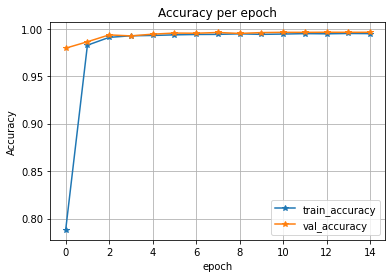


I


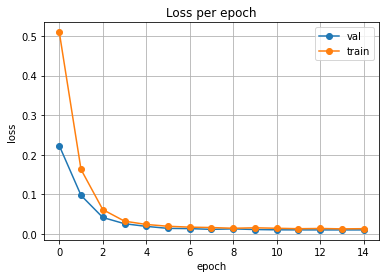


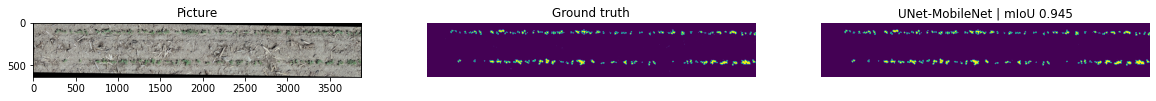

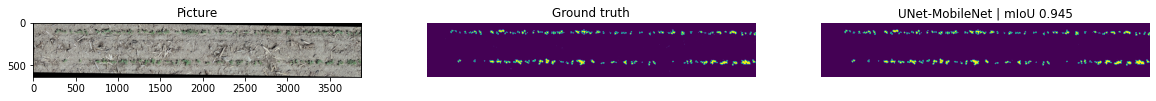

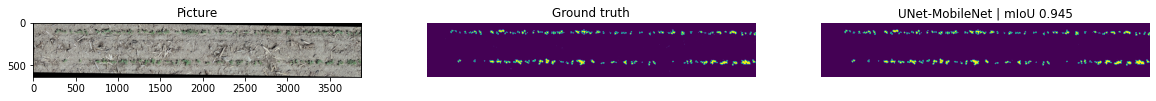


Fig. S11: Evaluation metrics results to perform the pixel-wise classification between soil and vegetation using the U-Net architecture with MobilenetV2 as the backbone.


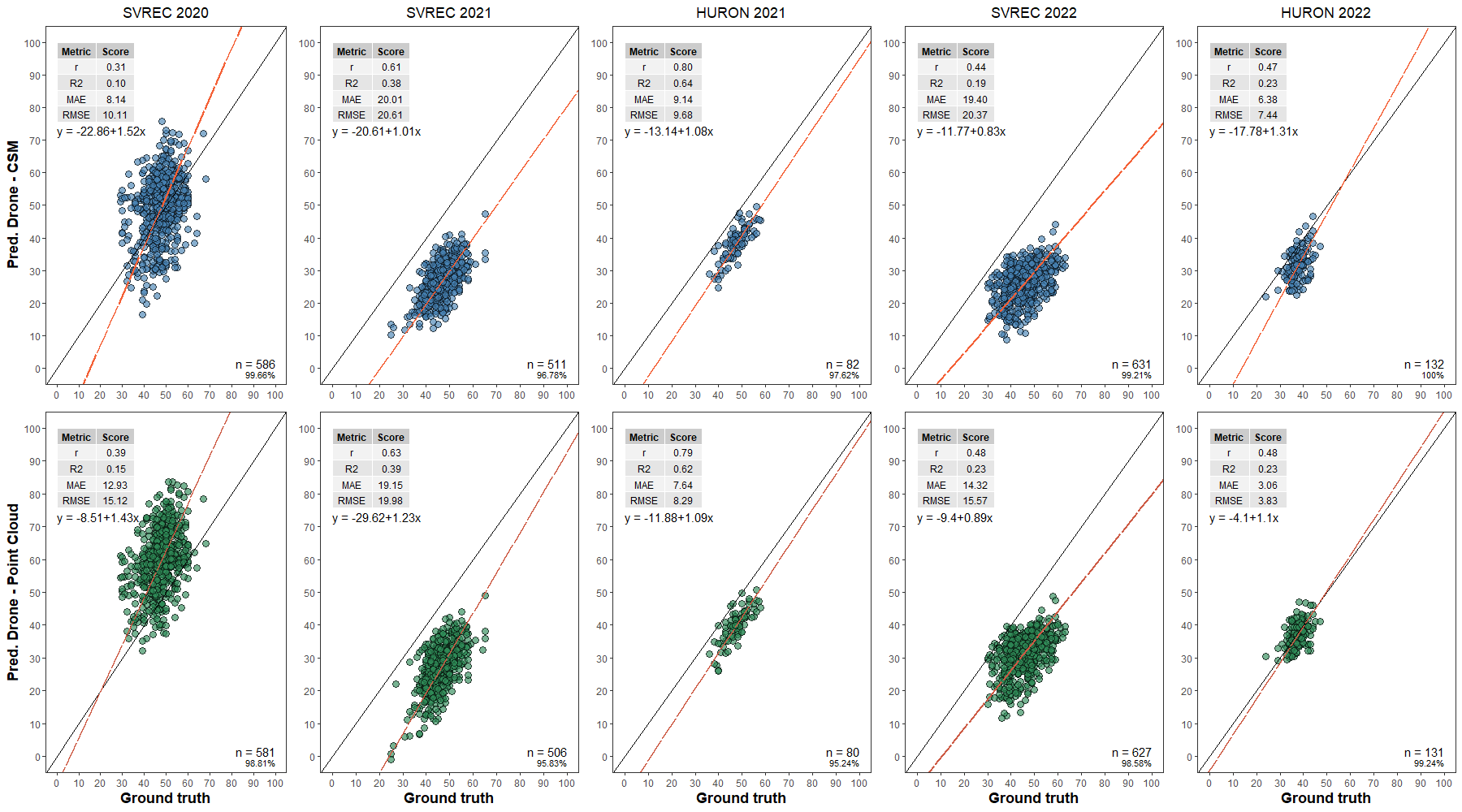


Fig. S12: Performance comparison between drone-based PH estimation methods using DSM/CSM and point cloud (PC) across five different environment datasets. The comparison is made under four evaluation metrics: Pearson's correlation coefficient (*r*), coefficient of determination (*r^2^*), Mean Absolute Error (*MAE*), and Mean Squared Error (*MSE*). The "n" value represents the total number of observations in each environment, while the percentage indicates the proportion of data points used after removing outliers.


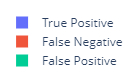

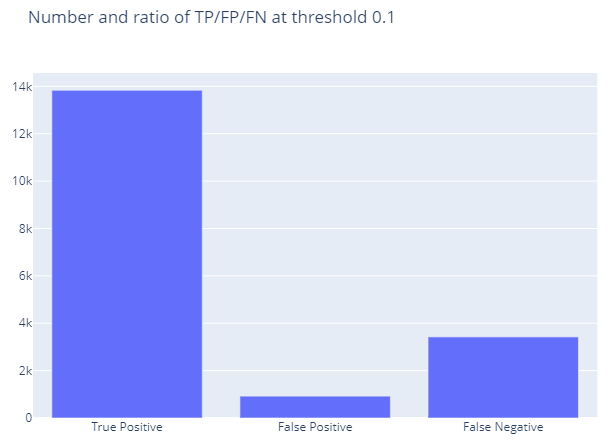

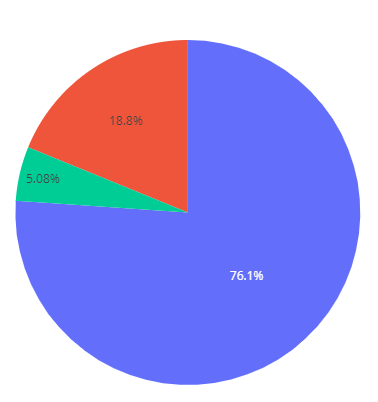


Fig. S13: Faster R-CNN performance model to identify early growth stage bean plants. True positive (TP) represents 76.1% (23529 plants detected), False Positive (FP) 5.08% (1570) and False Negative (FN) 18.8% (5813). Model metrics performance defined as: $Precision= \frac{TP}{TP+FP}\times100\%$, $Recall= \frac{TP}{TP+FN}\times100\%$, $F_{1}score= \frac{2x Precision \times Recall}{Precision+Recall}\times100\%$, and $Accuracy= \frac{TP}{TP+FP+FN}\times100\%$.

Table S1: Parameters used to perform plant height (PH) analysis in two locations and for three years using crop surface model (CSM) and digital surface model (DSM) or point clouds (PC) methods. qSoil represents the quantile values assigned for ground and non-ground values, and qVegetation is the quantile value used to extract the vegetation elevation.

| Env. | | Method | qSoil | qVegetation |
| --- | --- | --- | --- | --- |
| SVREC | 2020 | DSM | 0.50 | 0.90 |
|  | 2021 | CSM | - | 0.90 |
|  | 2022 | DSM | 0.25 | 0.99 |
| HURON | 2021 | CSM | - | 0.99 |
|  | 2022 | DSM | 0.25 | 0.99 |
|  |  |  |  |  |
| SVREC | 2020 | PC | 0.50 | 0.90 |
|  | 2021 | PC | 0.50 | 0.90 |
|  | 2022 | PC | 0.25 | 0.99 |
| HURON | 2021 | PC | 0.50 | 0.99 |
|  | 2022 | PC | 0.25 | 0.99 |

Fig S14. Radar chart illustrating the percentage distribution of the variables 2pl_dist (blue line, < 4 cm) and gap_plant (orange line, > 15 cm) across the evaluated plots, based on the specified intervals. The blue line indicates the percentage of plots with two or more plants spaced closely together (< 4 cm), while the orange line shows the percentage of plots with gaps between plants (> 15 cm). The average distance between plants was 8.45 cm, and the threshold distances (< 4 cm for closely spaced plants and > 15 cm for gaps) were set according to the desired plant distribution and population density per area.

Table S2. Plant distribution results across evaluated plots at SVREC 2022. For each measured plot (image_id), the stand count pipeline was applied to identify and count individual plants. The data was then adjusted to count the number of plants that are closely spaced within a plot (2pl_plot, < 4 cm) and the number of plots containing gaps between plants (gap_plant_plot, > 15 cm). The column plant_pop_total represents the total of plants counted within a plot.

| image_id | plant_pop_total | avg_dist_plot | 2pl_plot | gap_plant_plot |
| --- | --- | --- | --- | --- |
| 2201_1001_7m | 114 | 9.08 | 6 | 12 |
| 2201_1002_7m | 119 | 8.51 | 7 | 11 |
| 2201_1003_7m | 128 | 8.29 | 9 | 7 |
| 2201_1004_7m | 128 | 8.18 | 6 | 8 |
| 2201_1005_7m | 131 | 8.06 | 10 | 11 |
| 2201_1006_7m | 100 | 8.85 | 8 | 16 |
| 2201_1007_7m | 121 | 7.92 | 13 | 11 |
| 2201_1008_7m | 126 | 8.25 | 8 | 11 |
| 2201_1009_7m | 136 | 7.93 | 5 | 9 |
| 2201_1010_7m | 136 | 7.85 | 4 | 5 |
| 2201_1011_7m | 120 | 8.67 | 3 | 10 |
| 2201_1012_7m | 134 | 7.94 | 6 | 7 |
| 2201_1013_7m | 132 | 7.35 | 10 | 6 |
| 2201_1014_7m | 111 | 9.30 | 0 | 17 |
| 2201_1015_7m | 118 | 8.26 | 8 | 9 |
| 2201_1016_7m | 121 | 8.82 | 8 | 15 |
| 2201_1017_7m | 143 | 7.39 | 17 | 6 |
| 2201_1018_7m | 130 | 8.05 | 6 | 6 |
| 2201_1019_7m | 105 | 10.14 | 4 | 20 |
| 2201_1020_7m | 107 | 9.05 | 9 | 16 |
| 2201_1021_7m | 126 | 8.49 | 12 | 13 |
| 2201_1022_7m | 143 | 7.51 | 15 | 7 |
| 2201_1023_7m | 125 | 8.23 | 11 | 12 |
| 2201_1024_7m | 138 | 7.86 | 12 | 6 |
| 2201_1025_7m | 139 | 7.62 | 9 | 5 |
| 2201_1026_7m | 114 | 8.48 | 6 | 9 |
| 2201_1027_7m | 100 | 10.52 | 1 | 18 |
| 2201_1028_7m | 114 | 8.83 | 14 | 15 |
| 2201_1029_7m | 128 | 8.18 | 7 | 10 |
| 2201_1030_7m | 118 | 8.40 | 9 | 11 |
| 2201_1031_7m | 137 | 7.83 | 8 | 5 |
| 2201_1032_7m | 129 | 8.34 | 5 | 8 |
| 2201_1033_7m | 150 | 7.41 | 21 | 7 |
| 2201_1034_7m | 125 | 8.01 | 16 | 8 |
| 2201_1035_7m | 146 | 7.42 | 12 | 9 |
| 2201_1036_7m | 134 | 7.90 | 9 | 10 |
| 2201_2001_7m | 132 | 7.94 | 10 | 10 |
| 2201_2002_7m | 103 | 9.04 | 5 | 14 |
| 2201_2003_7m | 132 | 8.12 | 6 | 10 |
| 2201_2004_7m | 132 | 7.99 | 10 | 9 |
| 2201_2005_7m | 133 | 7.85 | 9 | 7 |
| 2201_2006_7m | 129 | 7.74 | 14 | 7 |
| 2201_2007_7m | 112 | 8.90 | 5 | 15 |
| 2201_2008_7m | 126 | 8.00 | 8 | 9 |
| 2201_2009_7m | 109 | 9.76 | 3 | 15 |
| 2201_2010_7m | 120 | 8.36 | 8 | 10 |
| 2201_2011_7m | 130 | 8.12 | 7 | 7 |
| 2201_2012_7m | 120 | 8.84 | 3 | 8 |
| 2201_2013_7m | 95 | 11.00 | 0 | 19 |
| 2201_2014_7m | 109 | 8.25 | 8 | 12 |
| 2201_2015_7m | 126 | 7.90 | 9 | 8 |
| 2201_2016_7m | 114 | 8.95 | 11 | 18 |
| 2201_2017_7m | 122 | 8.59 | 6 | 10 |
| 2201_2018_7m | 127 | 8.44 | 6 | 10 |
| 2201_2019_7m | 117 | 8.87 | 12 | 11 |
| 2201_2020_7m | 105 | 9.30 | 0 | 14 |
| 2201_2021_7m | 129 | 8.15 | 11 | 11 |
| 2201_2022_7m | 123 | 8.39 | 7 | 11 |
| 2201_2023_7m | 127 | 8.20 | 8 | 10 |
| 2201_2024_7m | 107 | 9.92 | 1 | 18 |
| 2201_2025_7m | 132 | 7.90 | 9 | 7 |
| 2201_2026_7m | 137 | 7.57 | 5 | 8 |
| 2201_2027_7m | 126 | 8.30 | 14 | 13 |
| 2201_2028_7m | 119 | 8.47 | 5 | 10 |
| 2201_2029_7m | 112 | 9.23 | 7 | 13 |
| 2201_2030_7m | 126 | 8.42 | 2 | 9 |
| 2201_2031_7m | 133 | 7.86 | 7 | 5 |
| 2201_2032_7m | 110 | 7.74 | 9 | 9 |
| 2201_2033_7m | 134 | 7.99 | 9 | 10 |
| 2201_2034_7m | 115 | 8.72 | 12 | 12 |
| 2201_2035_7m | 72 | 13.63 | 2 | 24 |
| 2201_2036_7m | 122 | 8.45 | 6 | 10 |
| 2202_1001_7m | 121 | 8.90 | 5 | 14 |
| 2202_1002_7m | 131 | 7.99 | 8 | 11 |
| 2202_1003_7m | 104 | 10.08 | 8 | 20 |
| 2202_1004_7m | 124 | 8.59 | 15 | 11 |
| 2202_1005_7m | 126 | 8.92 | 14 | 10 |
| 2202_1006_7m | 112 | 8.92 | 5 | 15 |
| 2202_1007_7m | 121 | 8.65 | 8 | 12 |
| 2202_1008_7m | 118 | 9.06 | 5 | 13 |
| 2202_1009_7m | 114 | 9.00 | 1 | 9 |
| 2202_1010_7m | 127 | 8.23 | 6 | 6 |
| 2202_1011_7m | 107 | 9.80 | 3 | 15 |
| 2202_1012_7m | 136 | 7.97 | 11 | 8 |
| 2202_1013_7m | 142 | 7.59 | 12 | 6 |
| 2202_1014_7m | 120 | 8.18 | 7 | 9 |
| 2202_1015_7m | 124 | 8.36 | 7 | 11 |
| 2202_1016_7m | 128 | 8.38 | 9 | 11 |
| 2202_1017_7m | 119 | 8.74 | 9 | 15 |
| 2202_1018_7m | 123 | 8.68 | 8 | 15 |
| 2202_1019_7m | 108 | 9.30 | 0 | 15 |
| 2202_1020_7m | 117 | 8.58 | 5 | 9 |
| 2202_1021_7m | 128 | 8.25 | 3 | 6 |
| 2202_1022_7m | 130 | 8.29 | 6 | 6 |
| 2202_1023_7m | 125 | 8.46 | 6 | 12 |
| 2202_1024_7m | 130 | 7.86 | 12 | 11 |
| 2202_1025_7m | 129 | 8.03 | 5 | 8 |
| 2202_1026_7m | 81 | 11.84 | 0 | 21 |
| 2202_1027_7m | 125 | 7.88 | 13 | 7 |
| 2202_1028_7m | 115 | 8.70 | 2 | 11 |
| 2202_1029_7m | 139 | 7.24 | 16 | 4 |
| 2202_1030_7m | 66 | 12.70 | 3 | 25 |
| 2202_2001_7m | 131 | 8.03 | 10 | 8 |
| 2202_2002_7m | 128 | 8.21 | 16 | 9 |
| 2202_2003_7m | 130 | 7.97 | 20 | 13 |
| 2202_2004_7m | 145 | 7.25 | 18 | 6 |
| 2202_2005_7m | 118 | 8.58 | 15 | 11 |
| 2202_2006_7m | 131 | 7.84 | 12 | 4 |
| 2202_2007_7m | 115 | 8.56 | 13 | 9 |
| 2202_2008_7m | 138 | 7.42 | 17 | 8 |
| 2202_2009_7m | 111 | 8.48 | 8 | 11 |
| 2202_2010_7m | 131 | 7.17 | 30 | 8 |
| 2202_2011_7m | 131 | 8.23 | 10 | 11 |
| 2202_2012_7m | 141 | 7.44 | 26 | 8 |
| 2202_2013_7m | 136 | 7.85 | 23 | 15 |
| 2202_2014_7m | 127 | 7.65 | 13 | 7 |
| 2202_2015_7m | 130 | 8.23 | 16 | 13 |
| 2202_2016_7m | 141 | 7.17 | 22 | 4 |
| 2202_2017_7m | 126 | 8.20 | 18 | 17 |
| 2202_2018_7m | 123 | 8.70 | 10 | 9 |
| 2202_2019_7m | 82 | 11.11 | 7 | 23 |
| 2202_2020_7m | 135 | 7.73 | 17 | 8 |
| 2202_2021_7m | 133 | 8.19 | 16 | 13 |
| 2202_2022_7m | 151 | 7.19 | 19 | 6 |
| 2202_2023_7m | 129 | 8.36 | 9 | 9 |
| 2202_2024_7m | 121 | 8.83 | 14 | 18 |
| 2202_2025_7m | 140 | 7.64 | 11 | 5 |
| 2202_2026_7m | 128 | 7.88 | 12 | 9 |
| 2202_2027_7m | 138 | 7.76 | 17 | 9 |
| 2202_2028_7m | 124 | 8.34 | 16 | 12 |
| 2202_2029_7m | 125 | 9.08 | 12 | 16 |
| 2202_2030_7m | 110 | 8.37 | 10 | 10 |
| 2202_3001_7m | 134 | 7.81 | 22 | 9 |
| 2202_3002_7m | 126 | 8.48 | 8 | 10 |
| 2202_3003_7m | 123 | 7.93 | 12 | 8 |
| 2202_3004_7m | 120 | 9.01 | 12 | 14 |
| 2202_3005_7m | 124 | 8.65 | 7 | 9 |
| 2202_3006_7m | 126 | 8.16 | 15 | 16 |
| 2202_3007_7m | 115 | 8.65 | 12 | 17 |
| 2202_3008_7m | 109 | 8.78 | 10 | 10 |
